# Brain tumor detection and segmentation: Interactive framework with a visual interface and feedback facility for dynamically improved accuracy and trust

**DOI:** 10.1371/journal.pone.0284418

**Published:** 2023-04-17

**Authors:** Kashfia Sailunaz, Deniz Bestepe, Sleiman Alhajj, Tansel Özyer, Jon Rokne, Reda Alhajj

**Affiliations:** 1 Department of Computer Science, University of Calgary, Alberta, Canada; 2 Department of Computer Engineering, Istanbul Medipol University, Istanbul, Turkey; 3 International School of Medicine, Istanbul Medipol University, Istanbul, Turkey; 4 Department of Computer Engineering, Ankara Medipol University, Ankara, Turkey; 5 Department of Health Informatics, University of Southern Denmark, Odense, Denmark; University of Engineering & Technology, Taxila, PAKISTAN

## Abstract

Brain cancers caused by malignant brain tumors are one of the most fatal cancer types with a low survival rate mostly due to the difficulties in early detection. Medical professionals therefore use various invasive and non-invasive methods for detecting and treating brain tumors at the earlier stages thus enabling early treatment. The main non-invasive methods for brain tumor diagnosis and assessment are brain imaging like computed tomography (CT), positron emission tomography (PET) and magnetic resonance imaging (MRI) scans. In this paper, the focus is on detection and segmentation of brain tumors from 2D and 3D brain MRIs. For this purpose, a complete automated system with a web application user interface is described which detects and segments brain tumors with more than 90% accuracy and Dice scores. The user can upload brain MRIs or can access brain images from hospital databases to check presence or absence of brain tumor, to check the existence of brain tumor from brain MRI features and to extract the tumor region precisely from the brain MRI using deep neural networks like CNN, U-Net and U-Net++. The web application also provides an option for entering feedbacks on the results of the detection and segmentation to allow healthcare professionals to add more precise information on the results that can be used to train the model for better future predictions and segmentations.

## Introduction

The brain is one of the major organs of human body. It controls most of the nervous system and it is responsible for managing most of the functions of our body [1]. The brain weighs about 3 pounds and it contains soft tissues, fat, protein, carbohydrate, water and salts [2]. The soft tissues (i.e. gray matter and white matter) contain neurons, blood vessels and other cells. The gray matter is the outer part of the brain having darker colors and the white matter is the inner part with lighter colors. This sequence is opposite for other major organ of the nervous system, the spinal cord. A tumor, “an abnormal mass of tissue” [3] may occur due to the deviation of regular cell life cycle or growth or both, may occur in the brain. Although the normal life cycle of a cell is that they grow, then are divided to two cells and eventually die, this cycle may be disrupted and some cells are divided into multiple cells uncontrollably and if they do not die they create a mass which is the tumor. Tumors can be benign (i.e. non-cancerous) or malignant (i.e. cancerous). Benign tumors do not invade other nearby tissues nor do they spread to other organs or parts of the body. Malignant tumors may, however, spread to other organs and invade nearby tissues. Cancer is the disease caused by such malignant tumors [4].

**Fig 1:**
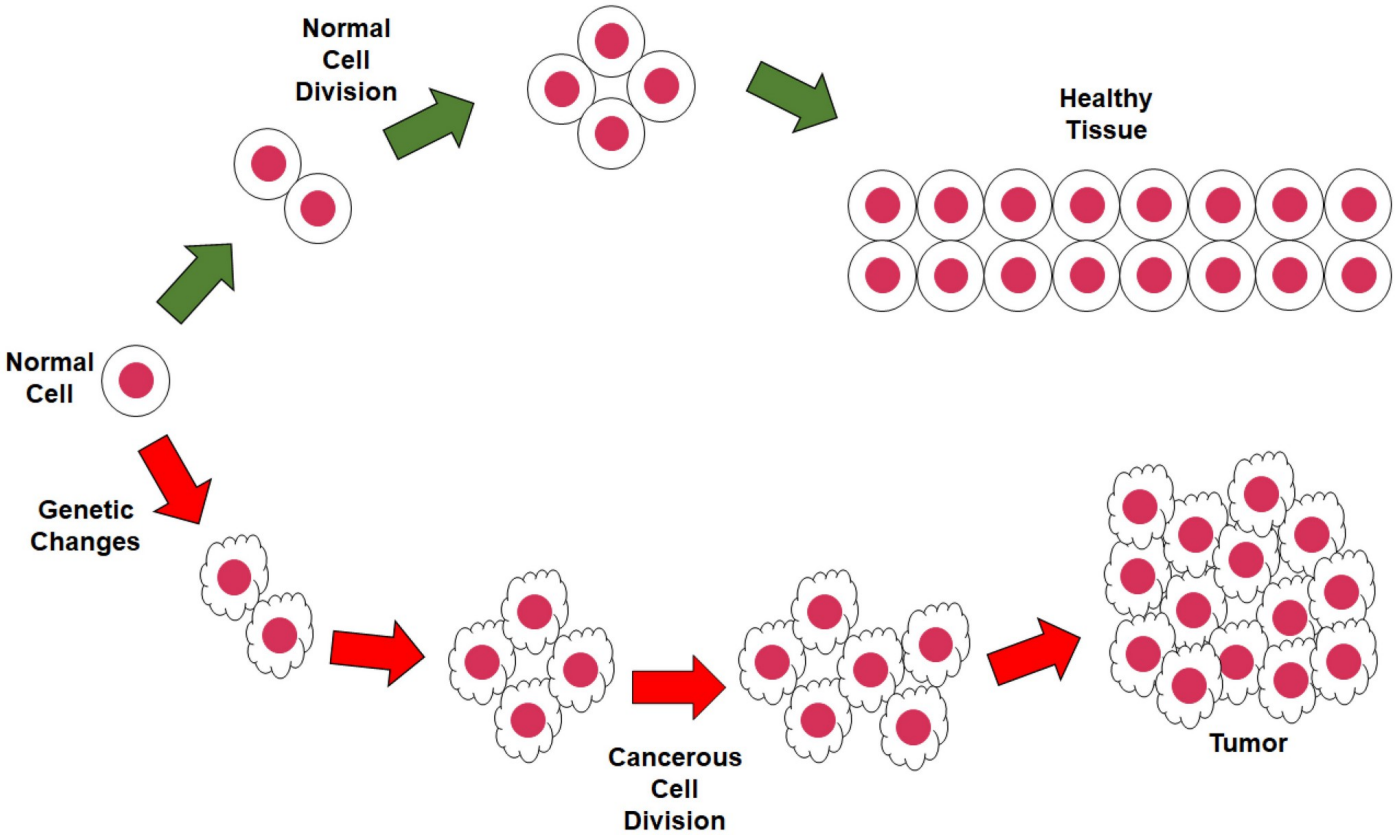
Sample image of brain tumor.

Brain tumors are tumors that starts in the brain or in the spinal cord [5]. They are called primary brain tumors if the origin of the tumor is brain or spinal cord. But, if the tumor originated in another part or organ and then spread to the brain then they are called secondary brain tumors or brain metastases. Fig 1 shows a sample of normal and abnormal cell growth for a brain tumor. Brain cancer, independent of how it originated, is one of the 10 deadliest cancers with a quite low 5 year relative survival rate of 32.5% [6]. 308,102 new brain cancer cases were documented in 2020 and 251,329 people died that year due to brain cancer worldwide [7]. More recent 2022 statistics for USA patients show that 700,000 people in USA are already suffering from brain cancer including 88,970 new primary brain tumor cases diagnosed and with the possibility of 18,200 deaths for due malignant tumor makes the relative survival rate only 36% [8, 9].

A brain tumor diagnosis includes different types of physical exams, blood tests, urine tests, medical images, biopsies and spinal taps [10]. Medical images are very popular non-invasive diagnosis tools that may include computed tomography (CT), positron emission tomography (PET) and magnetic resonance imaging (MRI) scans. The CT scan images are generated by combining X-rays taken from different angles to create a 3D view of an organ and it can also detect fluids (e.g., bleeding, blood vessels etc.) and bone structures, whereas PET scans take pictures of organs and tissues with the help of various mostly injected substances. MRI uses magnetic fields to generate details images of organs, tissues and they can also provide information about brain functionalities, chemical composition, blood flow etc. [11]. Although each type of imaging has its’ own benefits, MRI images are generally preferred for brain tumor imaging as they are less risky and produce clearer images. The different tissues of the brain is shown with various contrasts in different modalities of MRIs according to the imaging parameters like echo time, repetition time, flip angel etc. The four most common modalities for brain MRIs are T1-weighted (T1), T1-weighted contrast-enhanced (T1ce), T2-weighted (T2), and fluid attenuated inversion recovery (FLAIR) [12]. Generally, there are three anatomical positions for MRIs—axial, coronal and sagittal [13, 14] due to three different plane cross sections.

Medical professionals can asses the brain tumor, tumor location, tumor size, tumor area and other tumor properties from MRI images. Researchers have, however, been trying to automate those tasks due to the high cost of having this done by the medical professionals. Initially various conventional methods like thresholding/filtering, morphology-based models, geometry-based models, contouring, region-based models etc. were used for brain tumor detection and segmentation for automated brain tumor image analysis [15]. Once machine learning (ML) models became popular and showed higher efficiency in classification and image analysis tasks, researchers started to focus more on ML-based tumor detection and segmentation models using supervised, unsupervised and hybrid models [16]. With the emergence of more advanced artificial neural networks (ANN), deep neural networks (DNN) became more popular for the brain medical image analysis with deep learning (DL) models due to the high performance and accuracy of the outputs [17]. More recent transfer learning (TL) models and hybrid or ensemble models have also become quite popular in this research field [18].

Medical image analysis aims at detecting abnormalities from images and then extracting the abnormal region from the images. The first task in brain tumor analysis from brain medical images is therefore called ‘brain tumor detection’. The task is to detect if brain tumor is present in a brain medical image or not [19]. It can also be represented as an image classification problem where the input image can be classified as either a healthy image/non-tumor image or a tumor image. The second task of the major brain medical image analysis task is similarly ‘brain tumor segmentation’. After having identified that there is a tumor in the brain medical images, the next task is to divide the image into multiple segments or objects based on the similarity and dissimilarity between the different regions of the image. Brain tumor segmentation therefore focuses on segmenting or extracting only the tumor regions from the rest of the image for further analysis of tumor properties [20]. In this paper, a novel automated system to detect and segment brain tumors from brain medical images is proposed using DL-based approaches.

**Fig 2:**
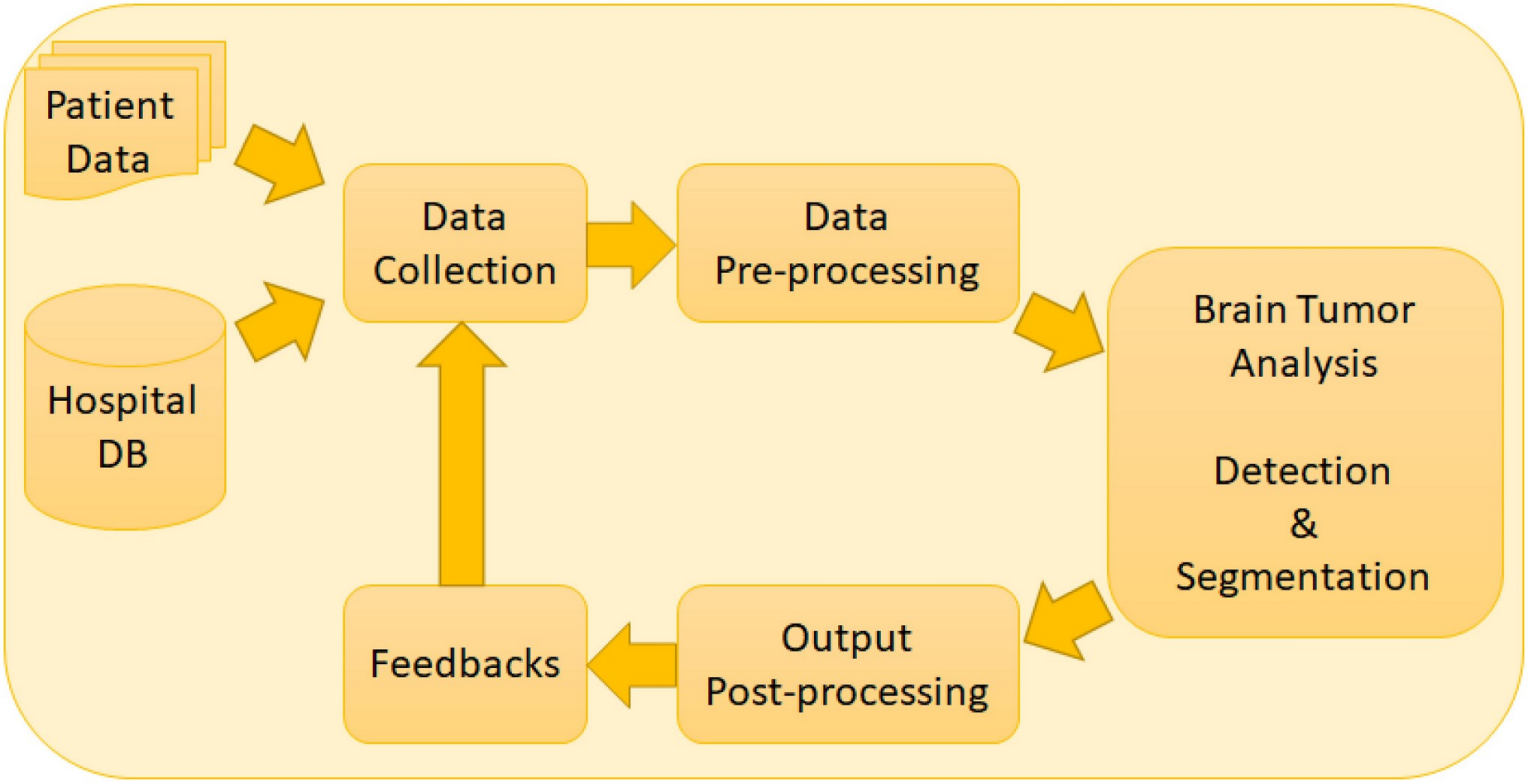
Proposed automated system for brain tumor analysis.

In our effort to serve healthcare professionals who deal with various types of tumors and infections, we initiated a project to develop a system having a visual interface for dealing with each type of diseases which is capable of identifying infected spots within an image (e.g., MRI, X-Ray, CT, etc.). Our target is to have a decision support system which increases the confidence of experienced professionals and new professionals in assessing MRI images. In this regards, we have already developed for COVID-19 an effective visual interface with feedback capabilities [21]. In this paper, we describe an automated system for brain tumor detection and segmentation from brain medical images. It is a web application which will help a healthcare professional with an initial screening of the images. The web application based system is able to provide decisions about the presence or absence of brain tumor in a brain medical image including a detection probability and provide a segmentation of the tumor from the brain imaging with a tumor confidence score, tumor area score and tumor ratio score. Fig 2 shows the architecture of the proposed system. The proposed model provides a web application that can be used to upload patient data by the user or it can directly access patient data from the connected hospital or medical databases. After the data collection process, the data is pre-processed and sent to the brain tumor analysis for brain tumor detection and segmentation. Then the generated outputs are pre-processed and the user has the option to provide feedbacks on the results as required.

**Fig 3:**
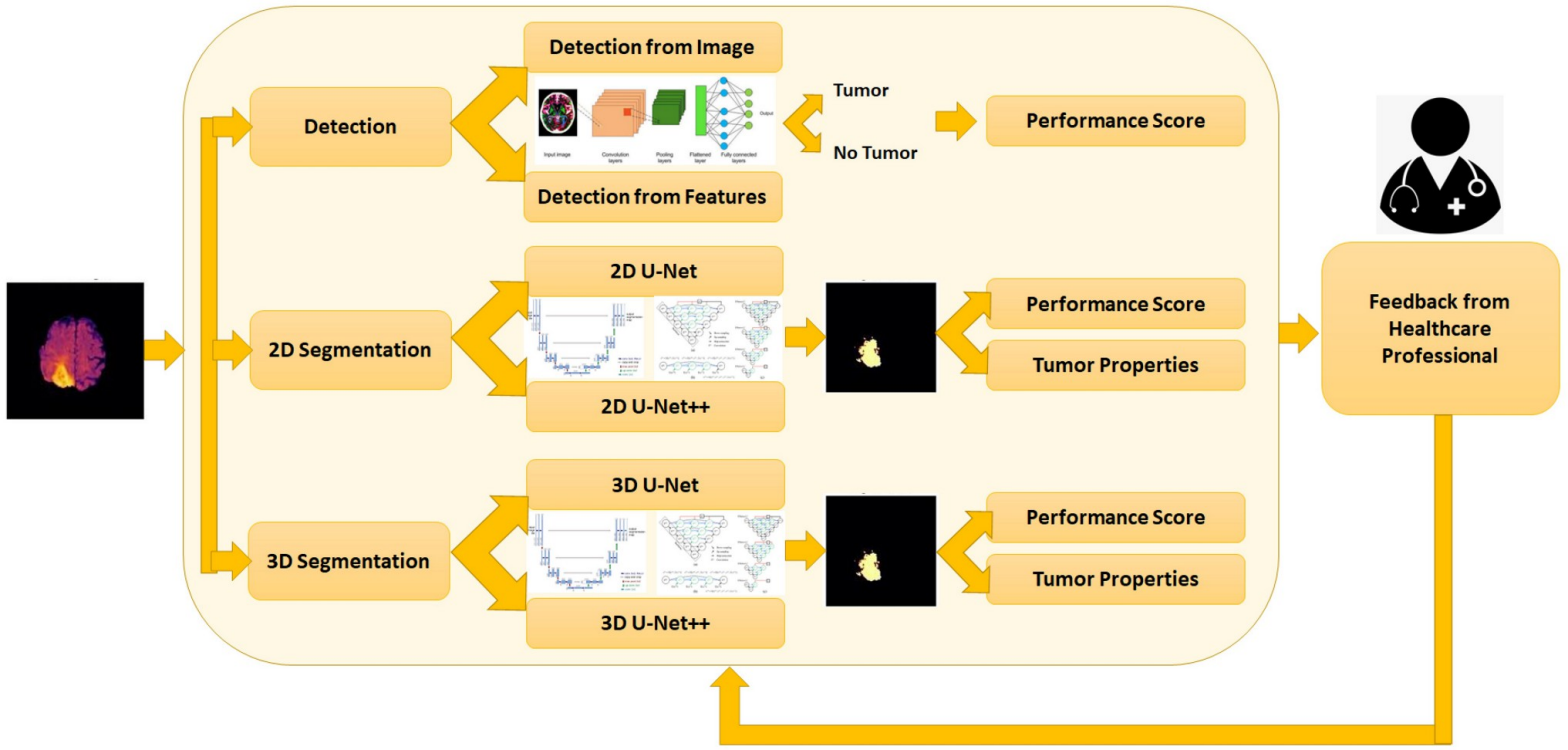
Overall framework for the proposed system.

Fig 3 shows the overall framework for the developed system in more details. Users can upload images (for tumor detection or 2D segmentation) or feature sets (for tumor detection) or nifti files (for 3D tumor segmentation) to the system based on the user requirement. The system provides the options for detection, 2D segmentation and 3D segmentation at the beginning. Then the users can specify if they want to use an image or feature sets for the detection part. For both 2D and 3D segmentation, the users have the choice of applying U-Net or U-Net++ model for the task. Based on the users choices and uploaded inputs, the system uses the saved trained models to predict and segment brain tumors from the user uploaded input data. Then the system provides output decisions (i.e. tumor or non-tumor) or segmentation information (i.e. the segmented tumor) with some performance scores for the users. The users or medical professionals can also provide their feedbacks on the output with the feedbacks being included in the training models to incorporate the professional feedback for future detection and segmentation.

The objectives and major contributions of this research are as follows -

- provide a complete web application for the medical professionals for two major brain tumor analysis tasks—tumor detection and tumor segmentation—that can be connected to existing medical databases for data access or that can operate independently using user input,
- deliver multiple types of options for each of the two tasks (i.e. detection with image input and detection with image features, 2D segmentation with U-Net and 2D segmentation with U-Net++, 3D segmentation with U-Net and 3D segmentation with U-Net++) for a more user-friendly application,
- provide medical professionals with the opportunity to review the outputs generated by the system and include their feedbacks to the application to improve the accuracy of the tasks that can be incorporated with the data for future executions.

The rest of the paper first provides brief summaries of the few related works on similar researches in the next Section. The methodology of our proposed system is explained after that. Then experimental results are included and finally some concluding remarks are provided.

## Related works

Although most recent brain medical image analysis systems use DL and ML models due to their high accuracy, a few researchers are still working on improving the conventional approaches such as thresholding, geometry, morphology, contouring etc. Nyo et al. [22] recently proposed a thresholding and morphology based model for brain tumor segmentation from brain MRIs. After converting the images into grayscale, they removed noise and applied Otsu’s thresholding algorithm to segment the tumor region from the MRI and then used opening and closing morphological operations for post-processing the segmented images. Their method was applied to 110 FLAIR images from BRATS 2015 [23] dataset for 2, 3, and 4 class models and achieved around 90% accuracy. Another recent thresholding and morphology based model applied a similar method for brain tumor segmentation from brain MRI, while adding a brain tumor severity detection (i.e. benign tumor or normal.) [24]. They created a dataset combining online datasets and gathering images from hospitals and then pre-processed the 2294 images for the threshold-based segmentation that segmented the tumor from the background. Then morphological operations were applied and a connected component analysis was done to get the solidity, the area and the bounding box of the tumor. The highest density area was extracted and checked with the maximum area of connected pixels. If both were same then the tumor was identified and the image was labeled as a ‘benign tumor’ image. Otherwise the image was considered as a normal/healthy image.

Most of the recent researches on brain tumor detection and segmentation apply conventional approaches as part of the pre-processing or post-processing methods for ML or DL hybrid or ensemble models. Support vector machine (SVM), random forest (RF), fuzzy C-means (FCM), K-means clustering are few of the popular ML models for tumor detection, segmentation and classification [25]. Hasanah et al. [26] recently proposed a ML-based brain tumor classification using filtering, contouring and thresholding as part of pre-processing and segmentation of the tumor. The MRIs were filtered with median filtering and the skulls were stripped from the images to use binary thresholding for a contouring algorithm. The largest contour was used for tumor feature (i.e. intensity and GLCM features) extraction. A SVM model used these features to classify the tumors into Glioma, Meningioma and Pituitary tumors with 95.83% average accuracy. A FCM clustering with level set method called fuzzy kernel level set (FKLS) was applied for 3D tumor segmentation in [27] using a combination of conventional and ML methods. They applied symmetry analysis to create the bounding box for the volume of interest (VOI) and then used FCM and level set methods to minimize the energy function for tumor segmentation. The proposed model showed high Dice score (i.e. 97.62%) on the BRATS 2017 [28] dataset.

Currently DL-based techniques are the most popular tools for brain tumor image analysis. Convolutional neural network (CNN), recurrent neural network (RNN), visual geometry group (VGG), ResNet, Inception, autoencoders, U-Nets and their variations are the most popular DL models for brain tumor detection, segmentation and classification [29]. Researchers have also tried to combine conventional method and ML models with DL-models to enhance the performance even more. Chattopadhyay et al. [30] proposed a DL model with a CNN framework and they tried to include ML method in the CNN for tumor/non-tumor detection. They applied SVM as the activation function at the last layer of the CNN and showed that the accuracy was only about 15% and they therefore moved to using a Softmax activation function thus achieving more than 99% accuracy. Another CNN-SVM hybrid model was used for brain tumor classification into benign and malignant tumor classes in [31]. After some basic image pre-processing and skull stripping, the tumor images were segmented using thresholding. Then a CNN model extracted the feature maps from the segmented images and the feature maps were fed into a SVM for the final classification. The hybrid CNN-SVM model was compared to the separate CNN and SVM models and outperformed both of them with more than 98% classification accuracy.

Another CNN-based tumor classification model was recently proposed by Ayadi et al. [32]. They applied a CNN model to three brain tumor datasets (i.e. Figshare [33], Radiopaedia [34], and REMBRANDT [35]) and classified the images into 2 (i.e. tumorous or normal), 3 (i.e. normal, low grade glioma (LGG), high grade glioma (HGG)), 4 (i.e. normal, astrocytoma (AST), oligodendroglioma (OLI), glioblastoma multiforme (GBM)), 5 (i.e. AST grade 2, AST grade 3, OLI grade 2, OLI grade 3, GBM), and 6 (i.e. normal, AST grade 2, AST grade 3, OLI grade 2, OLI grade 3, GBM) classes. Their model achieved overall accuracy of 90.35% without data augmentation and 93.71% with data augmentation which was comparable to the accuracy of similar CNN-based brain tumor classifiers. A CNN-based ensemble model was proposed in [36] with two stage ensemble model for best feature extractions to classify the brain MRIs into normal, meningioma, glioma and pituitary tumor classes. Three different brain MRI datasets were merged to create a collection of 10620 MRIs and they were used separately and together. They were pre-processed and fed into five pre-trained CNN models (i.e. VGG-19, EfficientNet-B0, Inception-V3, ResNet-50 and Xception) and five classifier (i.e. softmax, SVM, RF, K-nearest neighbor (KNN) and AdaBoost) for choosing the best feature extractor and best classifier respectively. Finally, the final classifier classified the MRIs into 4 classes with more than 99% accuracy. They created a python-based UI for the users to upload brain MRIs for classifying them in real-time and provide confidence percentages for each class.

A CNN variation U-Net [37] and its’ variations are very popular for brain tumor image analysis and brain tumor segmentation from MRIs. Ilhan et al. [20] proposed a U-Net based brain tumor segmentation model with tumor localization and enhancement models. After pre-processing the tumor regions were localized and enhanced by using the intensity of the pixels and the standard deviations from the image histogram to separate the tumors from the non-tumor regions. Then the U-net model was applied to segment the tumor and it achieved 0.85—0.94 Dice scores outperforming similar tumor segmentation models. The localization and enhancement of the ROI improved the feature extraction and training of the U-Net by a noticeable amount. Aghalari et al. [38] proposed a modified U-Net for brain tumor segmentation from BRATS 2018 dataset. 2D slices containing only background were discarded from the 3D data and then the rest of the slices were normalized from T1ce, T2 and FLAIR images. A two-pathway-residual (TPR) block structure was added to the U-Net to extract the global features as well as the local features from the images. The TPR block at every U-Net level sent the extracted global features for concatenation at the next level, hence enhancing the feature maps. The model was trained for segmenting tumors and achieved around 89% Dice scores that outperformed most similar works and was comparable to other DL models.

An image driven U-Net model was proposed in [39] for brain tumor segmentation for the BRATS 2018 dataset. As the first and last few slices of the 3D MRIs did not contain much information, only slices from 30th to 120th were used for the analysis. After cropping each slice from dimension 240 X 240 to 192 X 192 to crop out the background parts, the Watershed algorithm was used to separate the image into a tumor region and a non-tumor region and finally a Z-score normalization was done before feeding the images into the U-Net. The U-Net model was trained and tested and achieved more than 98% Dice scores for both LGG and HGG tumor segmentation. Das et al. [40] also worked on U-net but from a different perspective. They experimented on the learning parameters of U-Net for brain tumor segmentation to achieve the optimal results for BRATS 2017 and BRATS 2018 datasets. The input images were pre-processed and normalized with N4ITK bias field correction and then cropped into 192 X 192 slices to be fed into the U-net. Five different types of activation functions (i.e. Tanh, ReLU, leaky ReLU, parametric ReLU and ELU) were applied on the U-Net to segment the complete tumor, the core tumor and the enhancing parts with 97% to 99% accuracy. Their experiments with the activation functions, filter size, pooling, batch normalization and dropout showed better performance for ReLU activation function and average pooling.

U-Net++ [41] is an extension of the U-Net model that aims to improve the performance of medical image analysis that has been implemented by Hou et al. [42] for brain tumor segmentation. They applied basic data pre-processing on the BRATS 2018 and BRATS 2019 datasets by normalizing and clipping the slices and then merging all of the modalities together. Then the resulting U-Net++ model with a hybrid loss function of binary cross entropy (BCE) and Dice loss was trained and tested. Their proposed model achieved almost 89% Dice scores for segmenting the whole tumor. Another U-Net++ based ensemble model for Glioblastoma segmentation was proposed in [43]. This ensemble model used EfficientNet [44] for data pre-processing instead of the common pre-processing methods. A 3D EfficientNet was applied on each of the four MRI modalities of the BRATS 2021 dataset the and average was used for the classification step. Each MRI was sliced to create 2D images along the axial, coronal and sagittal planes and four modality images were concatenated. A separate 3D U-Net++ was applied on each plane to produce the segmentation and a majority voting model was applied to generate the final output with 90% Dice score for the final tumor segmentation.

nnU-Net [45], a new self-configuring DL model for biomedical image segmentation tasks is another popular DL model used in medical image analysis now a days. Luu et al. [46] proposed an extended nnU-Net for brain tumor segmentation in BRATS 2021 challenge. They replaced the batch normalization with group normalization using axial attention mechanism in decoder and applying double filters in the encoder of U-Net. The larger encoder helped to manage the large amount of data more appropriately. Their proposed model was able to achieve more than 90% Dice score with the extended nnU-Net model by modifying only three properties of the original nnU-Net. Axial attention was also used in [47] for brain tumor segmentation recently. They applied axial attention to extract and use both local and global semantic features from the MRIs more accurately for tumor sub-region segmentations with a hybrid loss function. They chose the 3D U-Net and added the axial attention mechanism at the decoder of the 3D U-Net. The proposed model was tested with BRATS 2019 and BRATS 2021 datasets and achieved more than 84% Dice score outperforming six U-Net based models proving the advantage of axial attention in accurate local and global pixel feature extraction. An ensemble model including DeepSeg, nnU-Net and DeepSCAN for segmenting brain tumor was proposed in [48] for BRATS 2022 challenge. Both DeepSeg and nnU-Net were inspired by the U-Net architecture and DeepSCAN was inspired by U-Net and DenseNet. The ensemble model combined these three DL models and applied an expectation-maximization method used for medical image segmentations called simultaneous truth and performance level estimation (STAPLE). Their ensemble model achieved more than 88% Dice scores outperforming all three individual models. Their performance ranking showed that among the three models, nnU-Net performed the best and DeepSeg achieved the lowest rank.

Vijay et al. [49] recently proposed an extended U-Net called SPP-U-Net by replacing the residual connection with attention blocks and spatial pyramid pooling (SPP). The attention blocks added at the levels of the decoder enhanced the features by incorporating more local pixel features with their global feature dependencies. The SPP blocks collected the information from all encoder layers to provide more specific data for reconstruction at the decoder. Their experiments on the BRATS 2021 dataset with variations of presence and absence of the SPP blocks and showed that the models achieved comparable results. The results with SPP were better in average and the best result was achieved with one SPP block with almost 87% Dice scores. A different U-Net based approach with transformers was applied in [50] for brain tumor segmentation task on BRATS 2019, BRATS 2020 and BRATS 2021 datasets. Their hybrid model combining CNN and transformers implemented the shifted window based swin transformer blocks that were able enhance the learning process and achieved 81.15% Dice score outperforming similar transformer-based brain tumor segmentation models. Lin et al. [51] also proposed a CNN-transformer hybrid model for brain tumor segmentation called CKD-TransBTS (i.e., clinical knowledge-driven brain tumor segmentation). Their dual-branch hybrid encoder was able to extract the correlations between different modalities of MRIs, extracted more precise features from the fusion of multimodal MRIs. They also added a hybrid transformer-CNN block for each encoder layer to calibrate the features better. They grouped the inputs into two categories—T1, T1Gd and T2, FLAIR and their proposed novel model achieved more than 90% Dice scores outperforming basic U-Net, U-Net++, transformer based U-Nets and similar networks. Generally, in recent BRATS dataset based brain tumor segmentation, part of the solution contains the segmentation of different tumor tissues like necrotic, edema etc. But few researchers also extracted other tumor properties like tumor area, volume, location etc. [52]. Recently, Nalepa et al. [53] proposed an end-to-end pipeline for tumor sub region segmentations for both pre and post operative data with DL and then computed the bidimensional and volumetric properties of the tumors with a new RANO (i.e., response assessment in neuro-oncology) computation. They also proposed an efficient manual annotation process and discussed their experiments on pre and post operative data and their proposed model was able to achieve comparable performances. Their research outputs provided a new direction for various brain tumor property extraction and keeping track of patients state before and after surgery. Some UI based researches mentioned below also included some tumor property computations.

Some researchers also worked on basic UI-based systems to automate the tumor detection process. Some of these researches focused on brain tumor/non-tumor detection or brain tumor type classification using basic ML or DL models with various image intensity and texture features. Abdullah et al. [54] worked on a Matlab simulator for tumor/non-tumor detection and tumor area segmentation using a cellular neural network. MRIs collected from KPJ Penang specialists were used to train the network with some modified templates for corner detection (i.e. template 1), edge detection (i.e. template 2) and hole filling (i.e. template 3). The templates helped to detect the presence or absence of a tumor in the uploaded image. A ML-based benign or malignant tumor detection UI was provided in [55] for brain MRIs. A median filter was used to pre-process the images and then a hybrid of Otsu binarization and K-means clustering was applied for segmenting the images. Thirteen intensity and GLCM features were then extracted from the segmented images to train a SVM model for classifying the image into benign or malignant tumor classes. The proposed model achieved about 100% accuracy in classification.

Boudjella et al. [56] proposed a KNN based prediction model implemented in a graphical user interface (GUI) for brain tumor detection. A dataset with tumor and non-tumor labeled images were used for six features extraction (i.e. mean, variance, standard deviation, entropy, skewness, kurtosis) which wa then used to train a KNN model for image classification. The model parameters were adjusted to get the optimal outputs with k values between 1 to 20. A GUI was developed where the users can enter six features, test size and k value for the KNN classifier. The GUI can then generate the prediction with more than 80% accuracy and display the relevant patient information. A similar web-based software for tumor classification that provides the UI options in both English and Turkish was proposed in [57]. They applied CNN with python AutoKeras libraries on T1-weighted brain MRIs to classify the input image into meningioma, glioma and pituitary tumors. The users can upload .jpeg, .jpg or .png T1-weighted brain MRIs to the system and the classification prediction appears as output with 94% to 98% prediction accuracy.

Khan et al. [58] also proposed a UI-based system with Matlab for brain tumor detection and classification with SVM for three classes—normal scan, benign tumor, malignant tumor. The input images were pre-processed and then first order (i.e. mean, standard deviation, entropy, kurtosis, skewness, energy) and second order (i.e. smoothness, contrast, homogeneity, correlation, inverse different moment (IDM)) features were collected from both benign and malignant training data. The users can upload an image to the UI and the features are extracted to generate the final decision on the tumor class. Very recently, a mobile application for tumor/non-tumor detection model was proposed in [59]. The image datasets were pre-processed and fed into a simple CNN model for tumor/non-tumor classification. The trained models was then used for the mobile application where a user can select image by taking a photo with a mobile camera or select an existing image to upload. While uploading the image, the user can crop the image to remove background and then the uploaded image is classified into tumor or non-tumor class with percentages and the higher percentage class is the prediction. The proposed model achieved more than 77% true positive (TP) and true negative (TN) rates.

There are further researches done in this field using other types of ML, DL, TL and hybrid models for brain tumor detection and segmentation from brain medical images which have other advantages and limitations. The researches that provide some type of UI have mostly worked on brain tumor detection or classification. Some of them applied tumor segmentation as part of their detection or classification, however, this was not the major output from their algorithms. Most of the existing UI based researches focused on one task with a limited scope and restricted input and output types.

## Methodology

The proposed system represents a complete interactive framework for achieving various brain MRI analysis tasks to assist medical professionals. Although the web application framework is designed in a way that it is capable of adding any trained ML and DL models for both the detection and segmentation tasks, some of the well-performed DL models are added to the application currently for detection and segmentation. More recent DL models will be added for the users in future to choose from for each task to provide them with more options for detection and segmentation tasks. The current implementation includes CNN models for the tumor detection tasks and U-Net and U-Net++ models for tumor segmentation tasks.

As mentioned earlier, in this paper, CNN models are used for brain tumor detection from brain MRIs and features collected from MRIs. Information shared in recent brain tumor image analysis reviews [17, 60] showed that despite the usage of newer DL models, CNN and its’ variations, and hybrid models containing CNN models are still widely used in medical image analysis researches. Another literature review [61] recently showed that CNN models had the highest amount (i.e. 32%) of researches that used DL methods like CNN, TL, Encoder-Decoder, HDL etc. for brain tumor images analysis. Similarly, U-Net has been broadly used for different medical image segmentation with high performances compared to previous DL models. Although there are various old and new DL models (i.e. attention-based models, LSTM, encoder-decoders, TL models, cascaded networks, etc.) used in literature, U-Net and variations of U-Nets are still few of the most popular DL models for brain tumor image analysis as mentioned in the recent brain tumor analysis literature reviews [61, 62]. Hence, the CNN, U-Net and U-Net++ models are chosen for implementation and experiments in this paper and more DL models will be added to the framework in future.

The proposed system contains a web application for the automation of tumor detection and tumor area segmentation from 2D and 3D images using a few DL models. The rest of this section includes more details on the image features, DL models (i.e. CNN, U-Net and U-Net++) and the algorithms used for the complete process of the proposed automated system.

### Image features

Various features can be extracted from the pixels of an image to understand their characteristics for further analysis. In this paper, we focused on the image intensity features, discrete wavelet transform (DWT) features, gray level co-occurrence matrix (GLCM) features and texture based features. These features are discussed as follows. First order histogram or image intensity based features depend on the pixel values of an image [63]. Four intensity features such as mean, variance, skewness and kurtosis are extracted for the analysis of images in this research. The mean value can be computed by summing up the pixel values and dividing the summation by the total number of pixels in the image. The variance value is an indication for how much the pixel values are spread out. The skewness refers to a measurement of the asymmetry of the pixel values in the histogram. Finally, the kurtosis represents the flatness or peakedness of the pixel values distribution in the histogram. The intensity values are normally calculated after converting the image into a grayscale image.

The GLCM features represent the frequency of different grayscale level combination occurring together [63]. The co-occurrence matrix computes the relative frequencies of the co-occurrences of the neighbor pixels. The contrast refers to the number of variations that exists in the image, dissimilarity represents the distance between the co-occurrences of two pixels based on their joint probability, whereas homogeneity represents the similarity and increases with low contrast. Angular second moment (ASM) is another measurement of homogeneity, the energy is the frequency of repetition of pixel pairs, and the correlation computes the grey level linear dependency of the image. The DWT transforms an image in order to reduce the dimension of the image by dividing it into four parts—low-low (LL), low-high (LH), high-low (HL) and high-high (HH) containing the low frequency sub-bands, horizontal features, vertical features and diagonal features respectively, that covers the full frequency spectrum of the original image [64]. The DWT-Coefficient represents the difference between the wavelet function and the analyzed signal of the image. A few other texture-based features like entropy, local binary pattern (LBP) and Haralick features were also used in this paper [63, 65]. The entropy computes the randomness of the pixels, LBP represents the texture of the image by thresholding neighbor pixels based on a specific pixel and the Haralick features provides the texture of the image from the normalization of the GLCM.

### DL models

The data pre-processing and few dimensions may need to be changed based on the input image dimension (i.e. 2D or 3D) for the CNN, U-Net and U-Net++ and are explained in the ‘Experimental Setup’, the basic structures of CNN, U-Net and U-Net++ are discussed here.

#### CNN

**Fig 4:**
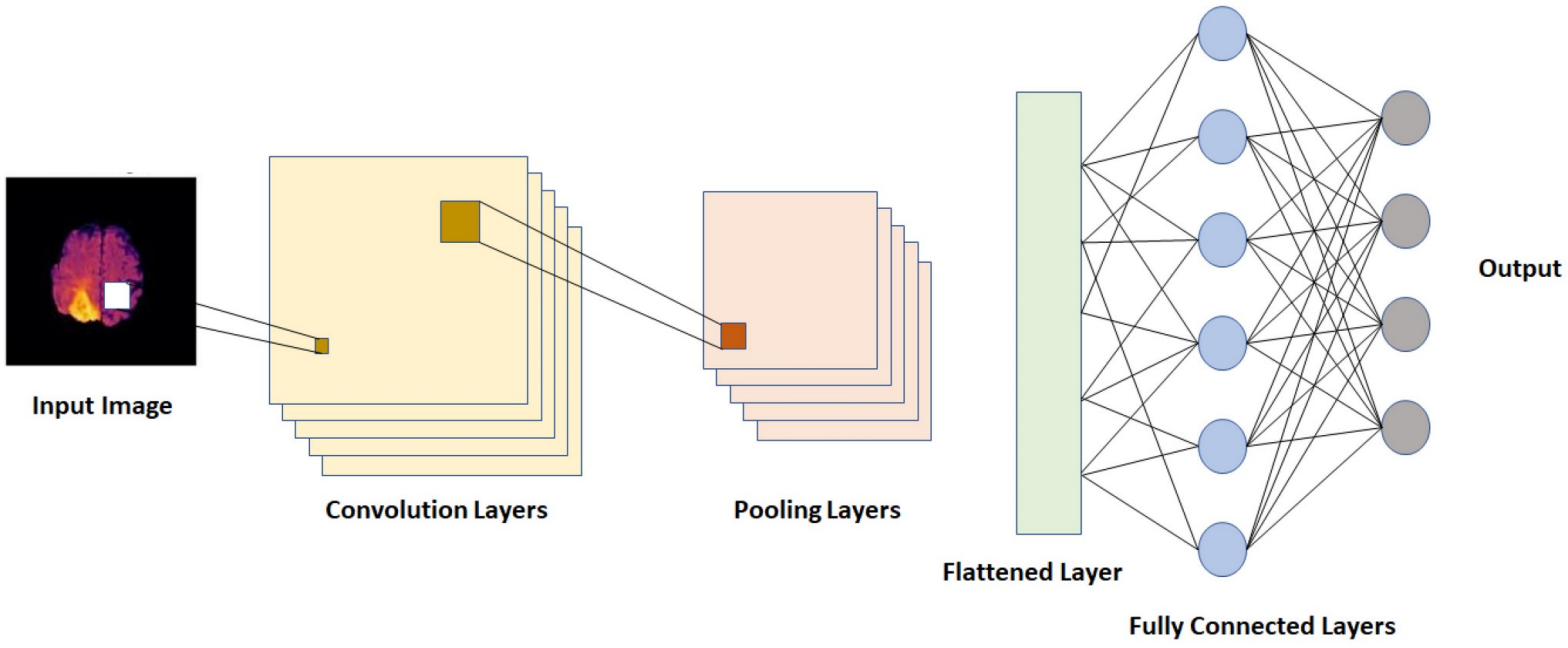
Basic CNN structure.

Convolutional neural networks (CNN) are among the most popular models for image classification, segmentation and analysis that have been developed over the last few years. The convolution layers of the model extracts different aspects of the features at each layer and incorporate these for an improved analysis of an image [66]. Medical images need more accurate feature extraction and an extensively well-trained feed-forward ANN to classify or segment pixels for output generation and for this CNN and its’ variations are the most frequently used models for medical image analysis tasks [67]. A CNN model uses a structure similar to the structure of our visual cortex (i.e. the primary region of our brain that receives and processes visual information [68]). The CNN model are trained on large image datasets with class labels in order to learn from the features automatically extracted at different convolution layers of the model and predict the labels for unknown test data based on the previously learnt patterns from training images. Fig 4 shows a sample CNN network with the basic few layers and nodes.

A CNN model reduces the total number of parameters by a large amount compared to a fully connected neural network. Instead of collecting data from the whole image at once, CNN scans the input image in blocks of n x n sliding windows (i.e. filter or kernel) at every convolution layer. The n x n block size is called the kernel size and it varies based on input and application type. The stride size is the number of pixels the sliding window moves at one step. The convolution reduces the dimension of the input without losing any important information in the image collected by the sliding window. So, at each convolution layer, a component-wise multiplication is done based on the kernel at every stride and the results are summed up to create the output for each pixel and finally the feature map is generated for the convolution layer. A pooling layer is applied after the convolution layer which has a similar operation as the convolution, except that it takes the average or maximum of the pixels to generate the feature map. So, it downsamples the output of the convolution layer with generally the same kernel size and stride size as the convolution layer. The number of convolution layers, neurons and pooling layers may vary based on the application. The number of hidden layers (i.e. depth of the network) can be varied and tested in order to find the optimal structure. In some cases some additional layers like batch normalization, dropout etc. may be added after each convolution [69]. The batch normalization is used to normalize the feature maps created for the next layer to make the computation faster and decrease the possibility of overfitting the model. A dropout layer is also added to avoid overfitting by randomly dropping out some neurons (i.e. setting the weight to zero). A flattened layer is used to convert the multi dimensional feature maps generated by the convolution layers into a one dimensional vector for the fully connected layer (i.e. a dense layer). Every neuron of the output of the fully connected layer is connected to each neuron of the input of that layer with different weights. An activation function is used in the dense layer to apply a non-linear transformation to generate the output by deciding which neurons should be activated during the transform.

A few more hyperparameters are used in CNNs like learning rate, loss function, optimizer, epochs, momentum, batch size etc. A loss function is a function that computes the differences between the target output and predicted output of the network to check the performance of the model. The goal is to achieve a minimum loss. The optimizer is a method to update the hyperparameters of the network to minimize the loss function and achieve optimal output. The learning rate is defined so as to control the amount of modifications introduced to the model hyperparameters to minimize loss. A higher learning rate can speed up the learning process of the model, however, it can lead to divergence and lower learning rate which slows down the learning process but gradually achieve convergence. The momentum decides the amount of changes needed based on the previous steps to avoid getting lost in local maxima by controlling the oscillation of the model. The epoch size represent the number of times the training model passes through the complete dataset and the batch size refers to the number of samples from the dataset passed through the network at a time. The hyperparameter selection is a crucial step for every DNN as the performance of the network depends largely on these hyperparameters.

#### U-Net

**Fig 5:**
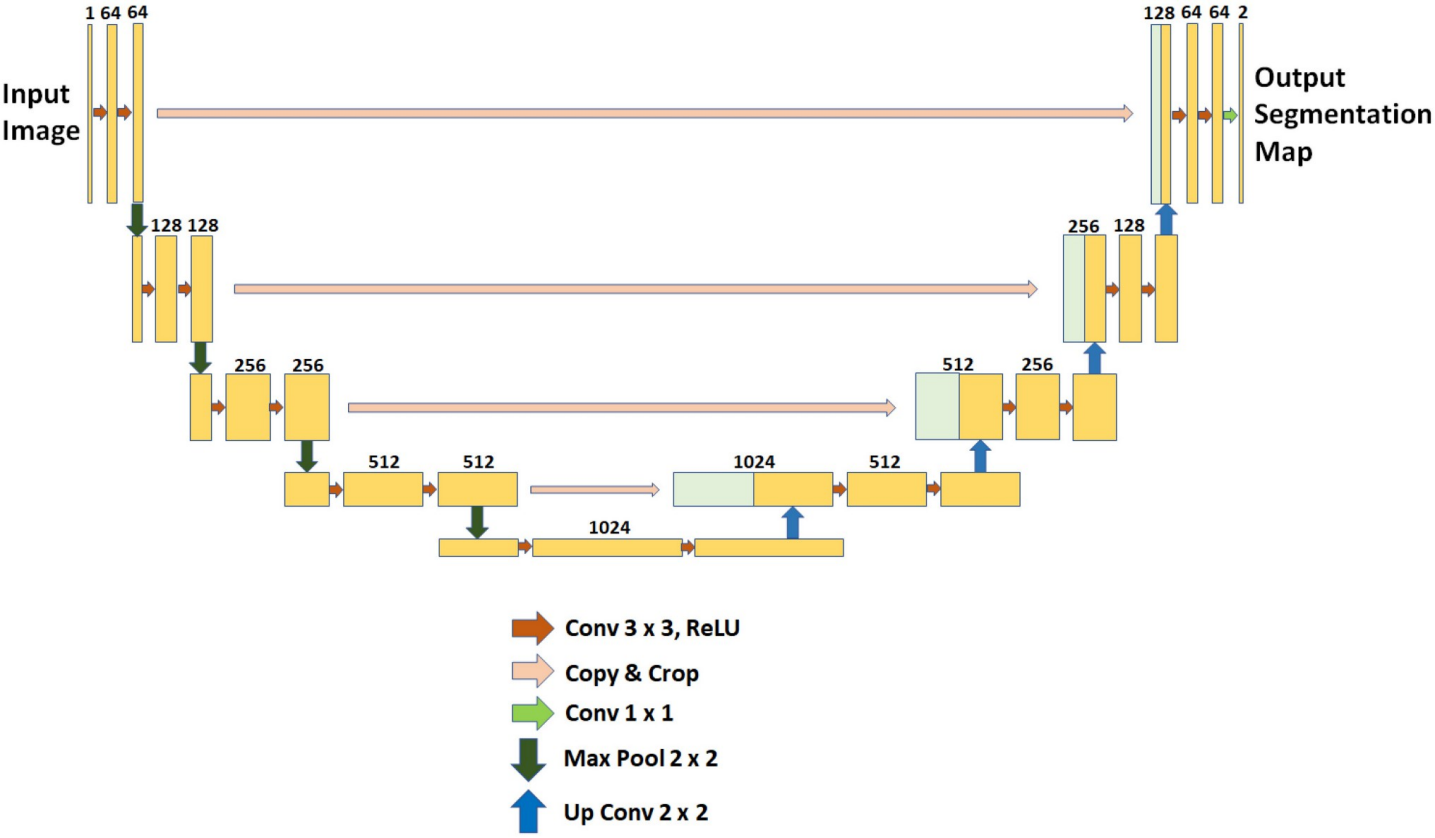
Basic U-Net structure.

U-Net is a variation of CNN specially proposed and developed for biomedical image segmentation and abnormality detection in medical images [37]. The U-Net is composed of a symmetrical U-shaped architecture containing a contracting path with convolution layers and an expansive path with transposed convolution layers represents the U-Net framework as shown in Fig 5. The contracting path has four levels of downsampling for the feature maps and the expansion path has four upsampling levels with a bridge to connect them. Each downsampling level includes two consecutive convolution layers for deep feature extraction and a max pooling layer to prepare the input for the next level. After the four downsampling levels, a bridge with just the two convolution layers is applied to pass the feature maps to the expansive path. Each level of the expansive path contains a similar structure but with a transposed convolution for feature extractions and two consecutive convolutions for upsampling after concatenating the feature maps generated by the transposed convolution and the corresponding feature map generated by the same level of the contraction path through skip connections. The concatenation is done to enhance the feature maps by combining the features of previous levels. The kernel size of U-Net is 3X3, the stride size is 2X2, the max pooling size is 2X2, the activation function used for all levels is a rectified linear unit (ReLU) and the output layer activation function is sigmoid. The hyperparameters are tuned or modified based on the type of application and inputs.

#### U-Net++

**Fig 6:**
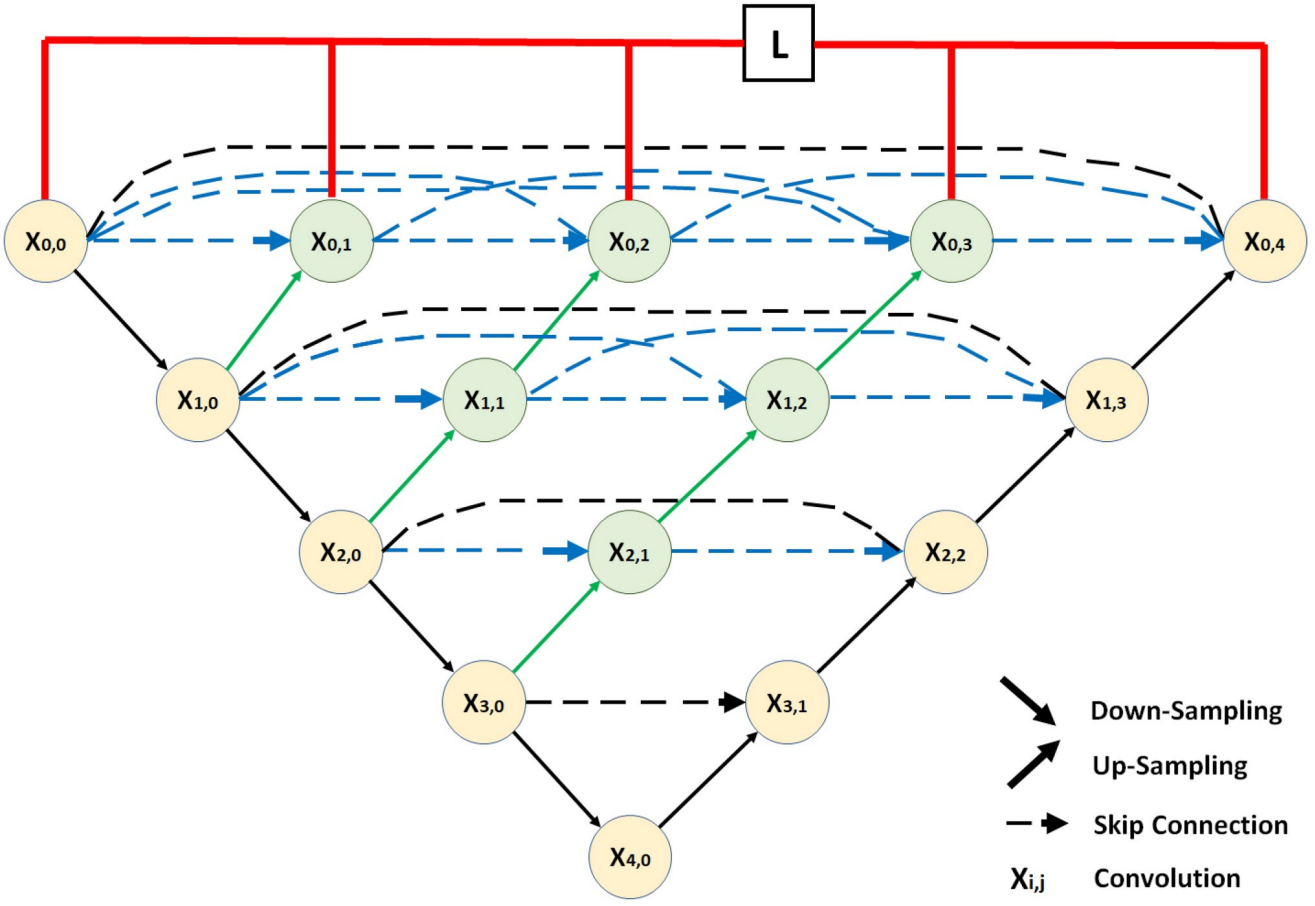
Basic U-Net++ structure.

U-net++, which is a variation of U-net to improve the medical image abnormality detection, was proposed in 2018 [41]. U-Net++ is a nested U-Net architecture containing more convolution layers on the skip paths to reduce the semantic gaps between the feature maps of the same level of contracting and expansive layers, additional skip connections and deep supervision for a denser U-Net structure. Fig 6 shows the basic U-net++ framework. The backbone of the U-Net++ (in black in Fig 6) is the basic U-Net structure, but the components on the skip pathways (in green and blue in Fig 6) and the deep supervision (in red in Fig 6) are the additions to the U-Net backbone which creates the U-Net++. The added convolution blocks on the skip pathways include a transposed convolution of the feature map used as input, then merges the feature map with the feature maps generated by the previous nodes and previous levels and then apply two consecutive convolution to upsample the feature map for the next node. The same computation is followed for all the nodes present in the skip pathway. As each node the feature maps from the previous nodes and levels are merged and the gap between the feature maps of the contracting and expansive path on the same level is reduced. As the nodes now have more similar feature maps, this structure improves the training time and performance. Unlike the U-Net structure, the deep supervision allows U-Net++ to combine the outputs from all branches to produce a more accurate prediction for the model. The hyperparameters used in the U-Net++ are the same as the hyperparameters that are used in the U-Net.

### Brain tumor analysis tasks

The two major brain tumor image analysis tasks are as note earlier in this paper: -i) brain tumor detection, and ii) brain tumor segmentation. Both of these tasks are implemented in our web application. The details of each task are now discussed.

#### Tumor detection

The tumor detection models uses the Kaggle dataset [70, 71] to train and test the models. The dataset includes images with names ‘Yxx’ or ‘Nxx’ where xx are numbers. ‘Y’ represents yes or tumor and ‘N’ refers to no or non-tumor. The first task before applying the detection models on the images is to clearly label each image for classification. Algorithm 1 shows the steps of the data labeling process. The images with tumors are labeled as 1 and the other images are labeled as 0 with the labels being stored in a file together with the image location. After labeling the images, some basic pre-processing steps are applied to the images. If the input image is a dicom file, then it is converted into a .jpg file for the further analysis. Then the input image is converted into a 2D format and resized into the dimension 256X256. Finally, the image is normalized so that the pixel scores are in the range 0 to 255. Algorithm 2 shows the image pre-processing steps for the tumor detection model.

**Algorithm 1** Tumor/non-tumor image labeling

**Require**: Input image, Image name

 *image*_*class* ← *image*_*name*[0]    ▹ Gets the first character of the image_name

 **if** image_class == ‘Y’ **then**

  *image*_*label* ← 1     ▹ 1 = Tumor

 **else**

  *image*_*label* ← 0    ▹ 0 = Non-Tumor

 **end if**

**Algorithm 2** Image pre-processing

**Require**: Input image

 **if** image_type == DICOM **then**

  *Convert dicom to jpg*

 **end if**

 *Convert image into* 2*D format*

 *Resize image into dimension* 256*X*256

 *Normalize each pixel score between* 0 *to* 255

The tumor detection model allows the user to either choose the image as input or the features as input for the tumor/non-tumor detection. Based on the user’s choice, the corresponding model is applied on the input to classify it into tumor or non-tumor classes and then calculating the evaluation scores as mentioned in Algorithm 3. If the user chooses the image as input, then the model trained with Algorithm 4 is applied and if the user choice is features the trained model from Algorithm 5 is used The tumor detection from image training model uses a simple CNN model for classifying the image by extracting deep features using the convolution layer. The feature maps extracted by the network are used to classify the image. The CNN model is trained with the pre-processed images and the image labels. The training model splits the dataset into training, validation and testing datasets. Then the ImageDataGenerator [72] process is used for data augmentation. The augmented data is then used to train the CNN model and the trained model is saved for future detection. The model is then tested with the test dataset. The model predicts a value and if the predicted value is greater than 0.5 then the test image is assigned to class 1, otherwise it is assigned to class 0. Class 1 refers to a tumorous image and class 0 refers to a healthy/non-tumorous image. The tumor detection from image features also uses a similar trained CNN model and trains the model with image features. The trained model is saved for the predictions of user inputs and follows similar steps as for the tumor detection process.

**Algorithm 3** Tumor/non-tumor detection

**Require**: Input image, Image features

 **if** user_choice == image **then**

  *Apply tumor*/*non* − *tumor detection from image trained model*

 **else**

  **if** user_choice == features **then**

   *Apply tumor*/*non* − *tumor detection from features trained model*

  **end if**

 **end if**

 *Provide detection output*

 *Generate evaluation scores*

**Algorithm 4** Model for tumor/non-tumor detection from image

**Require**: Pre-processed image, Image label

 *Split dataset into training*, *and testing set* (*i*.*e*. 80 : 20)

 *Split training set into training and validation set* (*i*.*e*. 80 : 20)

 *Apply image augmentation on training set using ImageDataGenerator*

 *Apply image augmentation on validation set using ImageDataGenerator*

 *Apply augmented data to train CNN model*

 *Save CNN model weights, performance scores*

 *Test trained model with testing dataset*

 **if** predicted_value > 0.5 **then**

  *test*_*class* ← 1

 **else**

  *test*_*class* ← 0

 **end if**

 **if** test_class == 1 **then**

  *test image is tumorous*

 **else**

  *test image is non* − *tumorous*

 **end if**

 *Generate classification report*

**Algorithm 5** Model for tumor/non-tumor detection from features

**Require**: Image name, Image features, Image label

 *Shuffle dataset randomly*

 *Split dataset into training*, *and testing set* (*i*.*e*. 80 : 20)

 *Split training set into training and validation set* (*i*.*e*. 80 : 20)

 *Apply data to train CNN model*

 *Save CNN model weights, performance scores*

 *Test trained model with testing dataset*

 **if** predicted_value > 0.5 **then**

  *test*_*class* ← 1

 **else**

  *test*_*class* ← 0

 **end if**

  **if** test_class == 1 **then**

 *test image is tumorous*

 **else**

  *test image is non* − *tumorous*

 **end if**

 *Generate classification report*

Algorithm 6 is used for extracting the features from the images. The input image is converted into 2D format and resized into the dimension 256X256. Then the intensity, DWT, GLCM, entropy, LBP and Haralick features are extracted separately. After saving each type of features against the image label, they are combined to have the complete feature set and saved with the corresponding image label. The features can be used separately or together to classify images as tumorous or non-tumorous.

**Algorithm 6** Feature extraction from image

**Require**: Input image, image Label

 *Convert image into* 2*D format*

 *Resize image into dimension* 256*X*256

 *Extract intensity features*

 *Extract DWT features*

 *Extract GLCM features*

 *Extract Entropy features*

 *Extract LBP features*

 *Extract Haralick features*

 *Save every feature against image*_*name*, *image*_*label*

 *Combine all features*

 *Save combined feature set against image*_*name*, *image*_*label*

#### Tumor segmentation

The tumor segmentation process is applied based on user choice. As mentioned in Algorithm 7, the user can choose between the 2D segmentation and 3D segmentation. If the user chooses 2D segmentation, then they can either U-net or U-Net++ when uploading the image. The same process is followed for 3D segmentation. The only difference is that the 3D segmentation requires four nifti files as inputs for T1, T2, T1ce and FLAIR modalities. The chosen model is applied on the input and the tumor segmentation with performance scores are shown as outputs.

**Algorithm 7** Tumor segmentation

**Require**: Input image (2D) or Input nifti files (3D)

 **if** user_choice == 2D segmentation **then**

  *Apply* 2*D segmentation image pre* − *processing*

  **if** user_sub_choice == U-Net **then**

   *Apply* 2*D U* − *Net tumor segmentation model*

  **else**

   **if** user_sub_choice == U-Net++ **then**

    *Apply* 2*D U* − *Net*++ *tumor segmentation model*

   **end if**

  **end if**

 **else**

  **if** user_choice == 3D segmentation **then**

   *Upload nifti files for T*1, *T*2, *T*1*ce and FLAIR modalities*

   *Apply* 3*D segmentation image pre* − *processing*

   **if** user_sub_choice == U-Net **then**

    *Apply* 3*D U* − *Net tumor segmentation model*

   **else**

    **if** user_sub_choice == U-Net++ **then**

     *Apply* 3*D U* − *Net*++ *tumor segmentation model*

    **end if**

   **end if**

  **end if**

 **end if**

 *Provide segmentation output*

 *Generate evaluation scores*

The 2D segmentation is applied after few basic pre-processing on the image as mentioned in Algorithm 8. If the input image is in dcom format, then it is converted into .jpg or .png format and transformed into grayscale. The dimensions are then resized into 512X512 and the pixels are normalized between 0 and 1. Then the image is expanded to 3D and downsampled to 128X128 and the chosen DL model is applied on it. The pre-processing in Algorithm 9 is the pre-processing needed for the 2D segmentation training model in the training and testing phase discussed in Algorithm 10. The training pre-processing is similar to the automated system input pre-processing with only the added tumor mask input and the tumor mask pre-processing required for the training.

**Algorithm 8** 2D segmentation image pre-processing

**Require**: Input image

 **if** image_type == DICOM **then**

  *Convert dicom to jpg*

 **end if**

 *Convert image into grayscale*

 *Resize image into dimension* 512*X*512

 *Normalize image pixels between* 0 *to* 1

 *Expand image to* 3*D by adding* 1 *as channel*

 *Downsample image to* 128*X*128

**Algorithm 9** 2D segmentation image pre-processing for training model

**Require**: Input image, tumor Mask

 *Extract*.*jpg files for MRI and tumor from*.*mat files*

 *Convert image into grayscale*

 *Resize image into dimension* 512*X*512

 *Normalize image pixels between* 0 *to* 1

 *Expand image to* 3*D by adding* 1 *as channel*

 *Expand tumor mask to* 3*D by adding* 1 *as channel*

 *Downsample image to* 128*X*128

 *Downsample image to‘mask to* 128*X*128

The training and testing models for U-Net and U-Net++ for 2D tumor segmentation shown in Algorithm 10. The pre-processed dataset and tumor masks are divided into training, validation and testing sets and then both the MRIs and tumor masks are flipped right and left and added to the training data for data augmentation. The updated training data is then processed by randomly changing the brightness level and zoom ranges to create random changes for data augmentation. The augmented data is used to train U-Net and U-net++ and the models are saved and tested to generate performance evaluations.

**Algorithm 10** Model for 2D brain tumor segmentation

**Require**: Pre-processed image, Tumor mask

 *Split dataset into training*, *and testing set* (*i*.*e*. 80 : 20)

 *Split training set into training and validation set* (*i*.*e*. 80 : 20)

 *Flipping training data*

 **for** *imageinimages*, *maskintumor*_*masks* **do**

  *rflip*_*image* ← *right*_*flip*(*image*)

  *rflip*_*mask* ← *right*_*flip*(*mask*)

  *lflip*_*image* ← *left*_*flip*(*image*)

  *lflip*_*mask* ← *left*_*flip*(*mask*)

 **end for**

 *training*_*image*_*new* ← *add*(*training*_*image*, *rflip*_*image*, *lflip*_*image*)

 *training*_*mask*_*new* ← *add*(*training*_*mask*, *rflip*_*mask*, *lflip*_*mask*)

 *Data generator for training data*

 **for** *random image in training*_*image*_*new*, *random mask in training*_*mask*_*new* **do**

  *bright*_*image* ← *brightness*_*range*(*image*)

  *bright*_*mask* ← *brightness*_*range*(*mask*)

  *zoom*_*image* ← *zoom*_*range*(*image*)

  *zoom*_*mask* ← *zoom*_*range*(*mask*)

 **end for**

 *Use augmented data for model training* & *validation*

 *Train U* − *Net*/*U* − *Net*++ *model*

 *Store model weights*, *evaluations*

 *Test trained model with testing dataset*

 *Provide segmentation output*

 *Generate evaluation scores*

The 3D segmentation process uses a very similar structure as the 2D segmentation process. The user input is pre-processed with Algorithm 11 then U-net or U-Net++ is applied to generate the tumor segmentation and performance scores, whereas Algorithm 12 is used to pre-process the input files and tumor masks for training the 3D U-Net and U-Net++ models as shown in Algorithm 13. For the 3D segmentations, the user needs to upload four nifti files, one for each modality (i.e. T1, T2, T1ce and FLAIR). The system then computes the mean and standard deviation for each of them and applies standardization on the images. As the first few slices and last few slices of the images do not contain much information, only the middle 70 slices from 155 slices in total (i.e. from slice 60 to 130) are stored for computation. Then they are resized to the dimension 128X128 and expanded. All four modality slices are then concatenated to create one 3D image for the DL models. After the U-Net or U-Net++ model is applied, the system generates the segmentation output with performance scores. The 3D segmentation training process uses the same training, validation and testing dataset divisions as the 2D process. After pre-processing the four modality files and the tumor mask files (according to Algorithm 12), the DL models are trained and tested following the steps in Algorithm 13.

**Algorithm 11** 3D segmentation image pre-processing

**Require**: Input nifti files for 4 modalities

 **for** *each modality file* **do**

  *mean*_*image* ← *mean*(*image*)

  *std*_*image* ← *std*(*image*)

  *standard*_*image* ← *standardization*(*mean*_*image*, *std*_*image*)

  **for** image_slice in range(60,130) **do**    ▹ Taking middle 70 slices from 155

   *Resize slice to* 128*X*128

   *Expand slice dimension*

  **end for**

 **end for**

 *preProcessed*_*image* ← *Concatenate* (*T*1, *T*2, *T*1*ce*, *FLAIR*)

**Algorithm 12** 3D segmentation image pre-processing for training model

**Require**: T1, T2, T1ce, FLAIR, Tumor mask

 **for** *each modality file* **do**

  *mean*_*image* ← *mean*(*image*)

  *std*_*image* ← *std*(*image*)

  *standard*_*image* ← *standardization*(*mean*_*image*, *std*_*image*)

  **for** image_slice in range(60,130) **do**    ▹ Taking the middle 70 slices from 155

   *Resize slice to* 128*X*128

   *Expand slice dimension*

  **end for**

 **end for**

 *mask*[*mask*! = 0] ← 1

 **for** mask_slice in range(60,130) **do**    ▹ Taking the middle 70 slices from 155

  *Resize mask*_*slice to* 128*X*128

  *Expand mask*_*slice dimension*

 **end for**

**Algorithm 13** Model for 3D brain tumor segmentation

**Require**: Pre-processed Image, Tumor Mask

 *Split dataset into training*, *and testing set* (*i*.*e*. 80 : 20)

 *Split training set into training and validation set* (*i*.*e*. 80 : 20)

 *Train U* − *Net*/*U* − *Net*++ *model*

 *Store model weights*, *evaluations*

 *Test trained model with testing dataset*

 *Provide segmentation output*

 *Generate evaluation scores*

The post-processing for the detection and segmentation tasks is simple and follows the steps in Algorithm 14. The detection post-processing simply computes the probability of the output class and shows that as the performance score. For the segmentation, the confidence score, the tumor area and the ration of the tumor compared to the brain area are calculated with the segmentation image output.

**Algorithm 14** Post-processing results

**Require**: Input image, Segmented tumor

 *Compute detection probility scores*

 *Compute segmentation confidence scores*

 *Compute tumor area*

 *Compute tumor ratio*

### Web application

**Fig 7:**
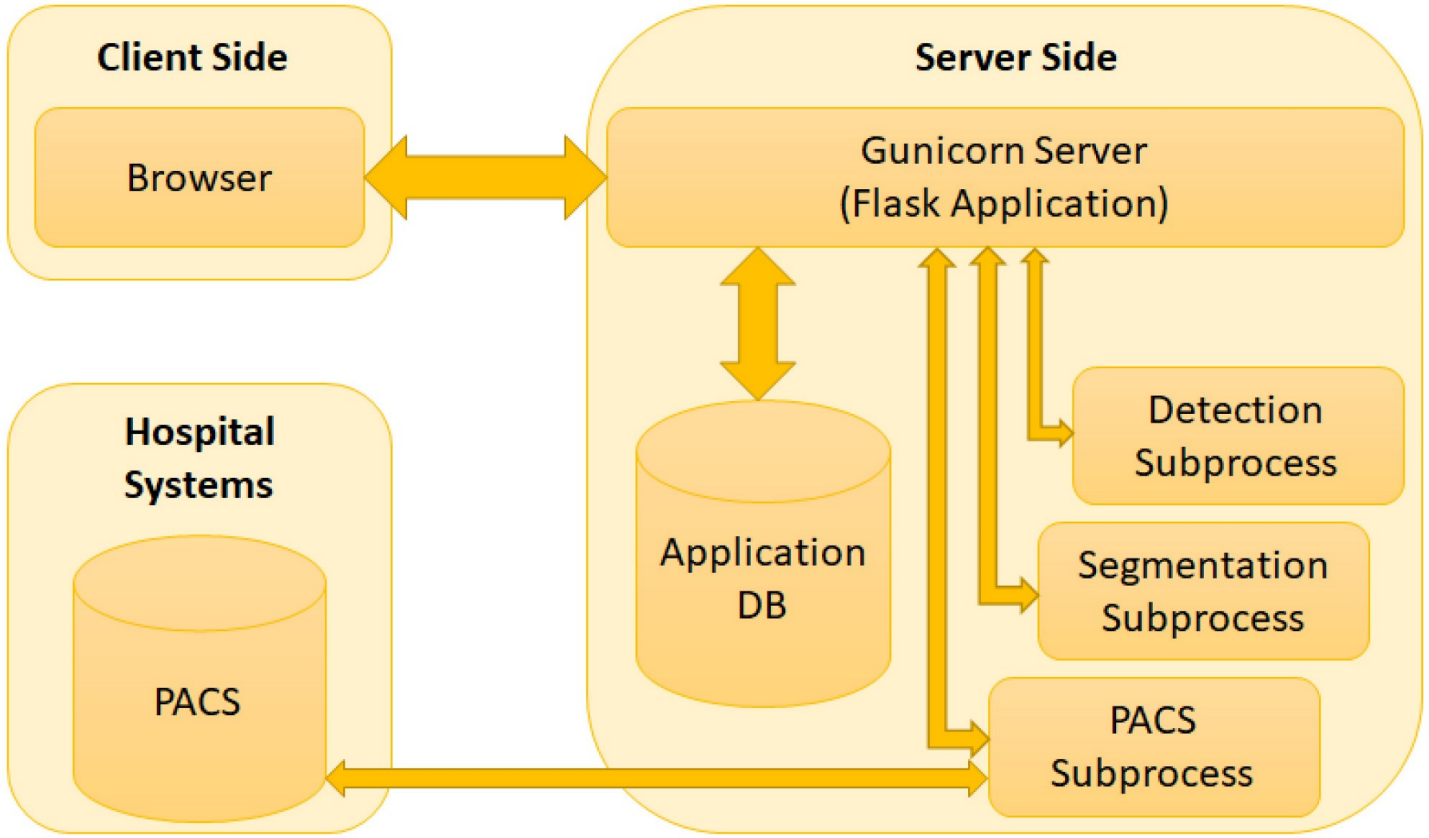
Web application architecture.

The web application developed for the proposed system can use user input (i.e. data uploaded by the user) or can access hospital data from a picture archiving and communication system (PACS). PACS can be integrated into the system and through that the system can connect to the imaging system of any hospital to access data. Any user or healthcare professionals can also use the browser to access the system from the client side. At the server side, a Gunicorn WSGI server is used to run the main Flask application and a PostgreSQL database (i.e. application DB) is used to store the data for the complete system. The detection, segmentation and PACS communication at the server side are designed as subprocesses so that they can be edited, added or removed easily. The subprocesses are independent, so they can use any programming languages or format without disrupting the main application. The PACS communication process uses C-ECHO request to create the communication with the PACS, C-FIND request to search for specific data, and C-MOVE request to request the selected medical images from the system. The Jinja2 [73] template engine with Flask is used to generate HTML content at the client side. The pages on the client side are generated by the Jinja2 template engine, whereas the PACS and feedbacks functionalities include additional content with HTML, Javascript and the asynchronous queries in PACS applied jQuery. Currently, the web application is deployed to a local development server (Intel Xeon Gold 6134 CPU, Nvidia P100 16GB GPU, 128GB RAM). The hospital systems (i.e., PACS) options are added to the proposed architecture to enable the medical professionals and/or institutes to incorporate our medical image analysis system into their existing databases, applications etc. The real-time usage of the proposed system in any healthcare organization will require some additional functionalities for the anonymization of patient information according to the rules and regulations of the organization and the ethical obligations of the state/country. The procedure of implementing these functionalities will depend on the conditions of the organizations and/or state/country, hence may vary for every organization. We will update the anonymization process according to these conditions during real-time usage of our proposed system. Fig 7 shows the web application architecture components and their connections between each other.

## Experimental results

The datasets, experimental setup for all models, the web application outputs and the model results are discussed in details in this section.

### Datasets

Different datasets were used to train and test the aforementioned models. The tumor/non-tumor detection models were trained and tested with the Kaggle tumor dataset [70, 71]. The dataset has both 2D and 3D images in .jpg, .jpeg and .png formats. The axial plane are visible in all 253 files. 155 of them are in the ‘Yes’ folder representing them as tumorous images and 98 of them are in the folder labeled ‘No’ referring to no tumors. The image dimensions and sizes are not consistent hence they need to be resized before they are used in this implementation. The 2D segmentation models were trained and tested with the CjData [33, 74, 75]. The dataset include 3064 T1-weighted contrast-enhanced MRIs of all three anatomical planes (i.e. axial, coronal, sagittal) from 233 patients suffering from three types of brain tumors—meningioma (708 images), glioma (1426 images) and pituitary tumors (930 images). The dataset contains 3064 .mat files each including patient ID, tumor type, tumor border, MRI and tumor mask (i.e. ground truth). The dataset was transformed by extracting the images and masks in 2D 512 X 512 images in .jpg format from the .mat files before applying it to the segmentation models. The 3D segmentation models used the most popular benchmark dataset for brain tumor image analysis—the BRATS dataset from the MICCAI brain tumor segmentation (BraTS) challenges [76]. In this paper, we applied the BRATS 2021 [77–82] dataset for our analysis. The BRATS 2021 training dataset contains 1251 folders in .nii .gz format. Each folder contains four 3D MRIs for the same patient for four modalities (i.e. T1, T2, T1ce and FLAIR) and one segmentation file (i.e. tumor ground truth) in the axial plane. The MRIs are in 3D nifti formats and they all have the size 240 X 240 X 155. Table 1 shows a summary of the datasets used in this paper.

**Table 1:** Datasets.

Task	Dataset	#Files	Labels	Type	Modality	Plane
Detection	Kaggle [70, 71]	253	Tumor Healthy	2D 3D	-	Axial
Segmentation	CjData [33, 74, 75]	3064	Meningioma Glioma Pituitary tumor	2D	T1	Axial Coronal Sagittal
Segmentation	BRATS 2021 [77–82]	1251	Glioma	3D	T1 T2 T1ce FLAIR	Axial

### Experimental setup

The DL models used for the tumor detection and segmentation are now discussed in detail. In all implemented models, the datasets were divided with a 80-20 distribution. 80% of the data was first separated and then the remaining 20% was kept as the testing data. Then the first 80% of the data was again divided with a 80-20 ratio to have 80% data as training data and the remaining 20% of the data as validation data. So, the test data fro all models were completely new to the trained models. Python [83] was used as the programming language to implement the tumor detection and segmentation models for this paper.

#### Tumor detection

The tumor detection task was implemented using a simple CNN with two different input types. In one implementation, the original brain MRI was used as input and the deep features were generated by the model for the detection task. In the other implementation, the image features were extracted beforehand from the MRI and the features were used as inputs to the CNN model for the final tumor/non-tumor classification task. For the CNN that used the image as input, we implemented a CNN with input size (256, 256, 3) and trained the model for 50 epochs. The first convolution layer was included with filter size 32, kernel size 8 X 8 and activation function ReLU. Then a dense layer with unit 32 and activation function ReLU was included. Then a 2 X 2 maxpooling layer and a dropout layer with a dropout of 0.2 was added. The second convolution layer had filter size 64, kernel size 8 X 8, activation function ReLU and was followed by a dense layer with 64 units and ReLU activation function, a 2 X 2 maxpooling layer and a dropout layer with 0.2 dropout. Lastly, a flatten layer was added before the final dense layer to generate the output. The final dense layer had unit size 1 and the sigmoid function as the activation function. The model used binary cross entropy as loss function and the RMSprop optimizer with a learning rate of 0.0001. The total number of parameters for the model was 352,609 where all of them were trainable. The CNN structure is shown in Table 2.

**Table 2:** CNN model structure for the tumor detection model with input images.

Layer	Type	Output Shape	Kernel Size
1	Convolution (ReLU)	(249, 249, 32)	8x8
2	Dense (ReLU)	(124, 124, 32)	-
3	Max Pooling	(124, 124, 32)	2x2
4	Dropout	(124, 124, 32)	-
5	Convolution (ReLU)	(117, 117, 64)	8x8
6	Dense (ReLU)	(117, 117, 64)	-
7	Max Pooling	(58, 58, 64)	2x2
8	Dropout	(58, 58, 64)	-
9	Flatten	215296	-
10	Dense (Sigmoid)	1	-

The CNN model that used the MRI features had a similar structure. In the first convolution layer with the ReLU activation function, the filter size was 64 and the kernel size was 2. Then a dense layer was added with 32 units with ReLU activation function followed by a dropout layer with 0.2 dropout. Then another dense layer with 16 units and ReLU activation function was added to the network. A maxpooling layer was included before another dropout layer with 0.2 dropout. Finally, a flatten layer was used before the final dense layer with unit size 1 and sigmoid activation function. There was 2,817 trainable and total parameters. The model was trained for 100 epochs with an Adam optimizer and a binary cross entropy loss function. Table 3 shows the details of the model structure. The Kaggle dataset [70, 71] and the features extracted from the dataset were used for the detection tasks.

**Table 3:** CNN model structure for the tumor detection model with input features.

Layer	Type	Output Shape	Kernel Size
1	Convolution (ReLU)	(3, 64)	2
2	Dense (ReLU)	(3, 32)	-
3	Dropout	(3, 32)	-
4	Dense (ReLU)	(3, 16)	-
5	Max Pooling	(1, 16)	2
6	Dropout	(1, 16)	-
7	Flatten	16	-
8	Dense (Sigmoid)	1	-

#### Tumor segmentation

The tumor segmentation was applied with four different models—2D U-net, 2D U-Net++, 3D U-Net and 3D U-Net++. Both of the 2D models had the same sets of hyperparameters and both of the 3D models applied the same sets of hyperparameters. The 2D U-Net model followed the U-Net structure [37] and the 2D U-Net++ model used the U-Net++ structure [41] as published. For the 2D U-Net, each layer of the contracting path included two consecutive convolution blocks with a kernel size 3 X 3, ReLU activation function and they were followed by a maxpooling layer with pool size 2 X 2 and stride size 2 X 2. Then a batch normalization with momentum 0.8 and a dropout layer with 0.1 dropout were added. The same structure was repeated for the complete contracting path and the filter sizes were 64, 128, 256, 512 respectively. The bridge layer between the encode and decoder had two consecutive convolution blocks with kernel size 3 X 3 and filter size 512. On the expansive path, the filter sizes were 512, 256, 128, 64 respectively. Each layer of the expansive path had a transposed convolution block with kernel size 3 X 3, stride size 2 X 2. After the concatenation layer, a dropout layer with 0.1 dropout was added. Finally, two consecutive convolution blocks with kernel size 3 X 3, activation function ReLU were included. The final output convolution layer of the 2D U-Net had filter size 1, kernel size 1 X 1 and activation function sigmoid. The model was tested with variations of parameter values and the optimal parameter set was used for training the model. The model was trained for 60 epochs with batch size 8 with an Adam optimizer as the optimizer function with a learning rate of 0.001. The U-Net++ layers had the same sets of hyperparameters as the 2D U-Net. Both models used a hybrid loss function that computed the Dice loss and binary cross entropy separately and then they were added together with 0.5 weight for each. The 2D U-Net model used total 22,718,529 parameters and 22,718,529 of them were trainable whereas 1,920 of them were non-trainable. Similarly, the 2D U-Net++ model applied 22,498,881 parameters in total (22,496,961 trainable and 1,920 non-trainable). Tables 4 and 5 show the structures of the models. The 2D segmentation models were applied to the CjData [33, 74, 75].

**Table 4:** 2D U-Net model structure for tumor segmentation.

Layer	Type	Output Shape	Kernel Size
0	Input Layer	(128, 128, 1)	-
1	Convolution (ReLU)	(128, 128, 64)	3x3
	Convolution (ReLU)	(128, 128, 64)	3x3
	Max Pooling	(64, 64, 64)	2x2
	Batch Normalization	(64, 64, 64)	-
	Dropout	(64, 64, 64)	-
2	Convolution (ReLU)	(64, 64, 128)	3x3
	Convolution (ReLU)	(64, 64, 128)	3x3
	Max Pooling	(32, 32, 128)	2x2
	Batch Normalization	(32, 32, 128)	-
	Dropout	(32, 32, 128)	-
3	Convolution (ReLU)	(32, 32, 256)	3x3
	Convolution (ReLU)	(32, 32, 256)	3x3
	Max Pooling	(16, 16, 256)	2x2
	Batch Normalization	(16, 16, 256)	-
	Dropout	(16, 16, 256)	-
4	Convolution (ReLU)	(16, 16, 512)	3x3
	Convolution (ReLU)	(16, 16, 512)	3x3
	Max Pooling	(8, 8, 512)	2x2
	Batch Normalization	(8, 8, 512)	-
	Dropout	(8, 8, 512)	-
5	Convolution (ReLU)	(8, 8, 512)	3x3
	Convolution (ReLU)	(8, 8, 512)	3x3
6	Transposed Convolution	(16, 16, 512)	3x3
	Concatenate (Layer 4 Convolution)	(16, 16, 1024)	-
	Dropout	(16, 16, 1024)	-
	Convolution (ReLU)	(16, 16, 512)	3x3
	Convolution (ReLU)	(16, 16, 512)	3x3
7	Transposed Convolution	(32, 32, 256)	3x3
	Concatenate (Layer 3 Convolution)	(32, 32, 512)	-
	Dropout	(32, 32, 512)	-
	Convolution (ReLU)	(32, 32, 256)	3x3
	Convolution (ReLU)	(32, 32, 256)	3x3
8	Transposed Convolution	(64, 64, 128)	3x3
	Concatenate (Layer 2 Convolution)	(64, 64, 256)	-
	Dropout	(64, 64, 256)	-
	Convolution (ReLU)	(64, 64, 128)	3x3
	Convolution (ReLU)	(64, 64, 128)	3x3
9	Transposed Convolution	(128, 128, 64)	3x3
	Concatenate (Layer 1 Convolution)	(128, 128, 128)	-
	Dropout	(128, 128, 128)	-
	Convolution (ReLU)	(128, 128, 64)	3x3
	Convolution (ReLU)	(128, 128, 64)	3x3
10	Convolution (Sigmoid)	(128, 128, 1)	3x3

**Table 5:** 2D U-Net++ model structure for tumor segmentation.

Layer	Type	Output Shape	Kernel Size
0	Input Layer	(128, 128, 1)	-
1	Convolution (ReLU)(d1)	(128, 128, 64)	3x3
	Convolution (ReLU)(d1)	(128, 128, 64)	3x3
	Max Pooling(p1)	(64, 64, 64)	2x2
	Batch Normalization(p1)	(64, 64, 64)	-
	Dropout(p1)	(64, 64, 64)	-
2	Convolution (ReLU)(d2)	(64, 64, 128)	3x3
	Convolution (ReLU)(d2)	(64, 64, 128)	3x3
	Max Pooling(p2)	(32, 32, 128)	2x2
	Batch Normalization(p2)	(32, 32, 128)	-
	Dropout(p2)	(32, 32, 128)	-
3	Convolution (ReLU)(d3)	(32, 32, 256)	3x3
	Convolution (ReLU)(d3)	(32, 32, 256)	3x3
	Max Pooling(p3)	(16, 16, 256)	2x2
	Batch Normalization(p3)	(16, 16, 256)	-
	Dropout(p3)	(16, 16, 256)	-
4	Convolution (ReLU)(d4)	(16, 16, 512)	3x3
	Convolution (ReLU)(d4)	(16, 16, 512)	3x3
	Max Pooling(p3)	(8, 8, 512)	2x2
	Batch Normalization(p3)	(8, 8, 512)	-
	Dropout(p3)	(8, 8, 512)	-
5	Convolution (ReLU)(d4)	(8, 8, 512)	3x3
	Convolution (ReLU)(d4)	(8, 8, 512)	3x3
6	Transposed Convolution(du 01)	(64, 128, 128)	3x3
	Concatenate[du 01,d1](du 01)	(128, 128, 128)	-
	Dropout(du 01)	(128, 128, 128)	-
	Convolution (ReLU)(c 01)	(64, 128, 128)	3x3
	Convolution (ReLU)(c 01)	(64, 128, 128)	3x3
7-1	Transposed Convolution(du 11)	(128, 64, 64)	3x3
	Concatenate[du 11,d2](du 11)	(256, 64, 64)	-
	Dropout(du 11)	(256, 64, 64)	-
	Convolution (ReLU)(c 11)	(128, 64, 64)	3x3
	Convolution (ReLU)(c 11)	(128, 64, 64)	3x3
7-2	Transposed Convolution(du 02)	(64, 128, 128)	3x3
	Concatenate[du 02,du 01,d1](du 02)	(256, 128, 128)	-
	Dropout(du 02)	(256, 128, 128)	-
	Convolution (ReLU)(c 02)	(64, 128, 128)	3x3
	Convolution (ReLU)(c 02)	(64, 128, 128)	3x3
8-1	Transposed Convolution(du 21)	(256, 32, 32)	3x3
	Concatenate[du 21,d3](du 21)	(512, 32, 32)	-
	Dropout(du 21)	(512, 32, 32)	-
	Convolution (ReLU)(c 21)	(256, 32, 32)	3x3
	Convolution (ReLU)(c 21)	(256, 32, 32)	3x3
8-2	Transposed Convolution(du 12)	(128, 64, 64)	3x3
	Concatenate[du 12,c 11,d2](du 12)	(384, 64, 64)	-
	Dropout(du 12)	(384, 64, 64)	-
	Convolution (ReLU)(c 12)	(128, 64, 64)	3x3
	Convolution (ReLU)(c 12)	(128, 64, 64)	3x3
8-3	Transposed Convolution(du 03)	(64, 128, 128)	3x3
	Concatenate[du 03,c 02,c 01,d1](du 03)	(256, 128, 128)	-
	Dropout(du 03)	(256, 128, 128)	-
	Convolution (ReLU)(c 03)	(64, 128, 128)	3x3
	Convolution (ReLU)(c 03)	(64, 128, 128)	3x3
9-1	Transposed Convolution(u 31)	(512, 16, 16)	3x3
	Concatenate[u 31,d4](u 31)	(1024, 16, 16)	-
	Dropout(u 31)	(1024, 16, 16)	-
	Convolution (ReLU)(c 31)	(256, 16, 16)	3x3
	Convolution (ReLU)(c 31)	(256, 16, 16)	3x3
9-2	Transposed Convolution(u 22)	(256, 32, 32)	3x3
	Concatenate[u 22,c 21,d3](u 22)	(768, 32, 32)	-
	Dropout(u 22)	(768, 32, 32)	-
	Convolution (ReLU)(c 22)	(256, 32, 32)	3x3
	Convolution (ReLU)(c 22)	(256, 32, 32)	3x3
9-3	Transposed Convolution(u 13)	(128, 64, 64)	3x3
	Concatenate[u 13,c 12,c 11,d2](u 13)	(512, 64, 64)	-
	Dropout(u 13)	(512, 64, 64)	-
	Convolution (ReLU)(c 13)	(128, 64, 64)	3x3
	Convolution (ReLU)(c 13)	(128, 64, 64)	3x3
9-4	Transposed Convolution(u 04)	(64, 128, 128)	3x3
	Concatenate[u 04,c 03,c 02,c 01,d1](u 04)	(320, 128, 128)	-
	Dropout(u 04)	(320, 128, 128)	-
	Convolution (ReLU)(c 04)	(64, 128, 128)	3x3
	Convolution (ReLU)(c 04)	(64, 128, 128)	3x3
10	Concatenate[c 01,c 02,c 03,c 04]	(256, 128, 128)	-
11	Convolution (Sigmoid)	(128, 128, 1)	1x1

The 3D U-Net and 3D U-Net++ models used the BRATS 2021 [77–82] dataset and they followed the same structure as the original U-Net and U-net++, with some additional modifications which were done to adjust the dimension changes for the 3D images. Again, the same sets of hyperparameters were used for the 3D U-net and the 3D U-Net++ models. The input size for the models were (128 X 128 X 4). Each layer of the contracting path had two convolution layers with kernel size 3 X 3, activation function ReLU where each convolution layer was followed by a batch normalization layer with a momentum of 0.8. Then the contracting layer had a final maxpooling with pool size 2 X 2 to generate the input feature map for the next layer. The filter sizes of the contracting layer were 64, 128, 256, 512 respectively. The bridge layer between the contracting and expansive path had a filter size 1024, kernel size 3 X 3 and ReLU activation function. Each layer of the expansion path had a transposed convolution layer with kernel size 2 X 2 and stride size 2 X 2. After the concatenation, there were two consecutive convolution layers with kernel size 3 X 3, activation function ReLU and each of them was followed by a batch normalization layer. The filter sizes for the expansive path were 512, 256, 128 and 64 respectively. The final convolution layer of the model had filter size 1, kernel size 1 X 1 and used the sigmoid activation function. The 3D models were trained for 50 epochs with batch size 8, Adam optimizer, 0.0001 learning rate and the Dice loss function. The 3D U-Net and 3D U-Net++ models had 31,055,873 (31,044,097 trainable and 11,776 non-trainable) and 24,266,977 (24,266,737 trainable and 240 non-trainable) parameters in total respectively. Tables 6 and 7 show the structures of the models.

**Table 6:** 3D U-Net model structure for tumor segmentation.

Layer	Type	Output Shape	Kernel Size
0	Input Layer	(4, 128, 128)	-
1	Convolution (ReLU)	(64, 128, 128)	3x3
	Batch Normalization	(64, 128, 128)	-
	Convolution (ReLU)	(64, 128, 128)	3x3
	Batch Normalization	(64, 128, 128)	-
	Max Pooling	(64, 64, 64)	2x2
2	Convolution (ReLU)	(128, 64, 64)	3x3
	Batch Normalization	(128, 64, 64)	-
	Convolution (ReLU)	(128, 64, 64)	3x3
	Batch Normalization	(128, 64, 64)	-
	Max Pooling	(128, 32, 32)	2x2
3	Convolution (ReLU)	(256, 32, 32)	3x3
	Batch Normalization	(256, 32, 32)	-
	Convolution (ReLU)	(256, 32, 32)	3x3
	Batch Normalization	(256, 32, 32)	-
	Max Pooling	(256, 16, 16)	2x2
4	Convolution (ReLU)	(512, 16, 16)	3x3
	Batch Normalization	(512, 16, 16)	-
	Convolution (ReLU)	(512, 16, 16)	3x3
	Batch Normalization	(512, 16, 16)	-
	Max Pooling	(512, 8, 8)	2x2
5	Convolution (ReLU)	(1024, 8, 8)	3x3
	Batch Normalization	(1024, 8, 8)	-
	Convolution (ReLU)	(1024, 8, 8)	3x3
	Batch Normalization	(1024, 8, 8)	-
6	Transposed Convolution	(512, 16, 16)	2x2
	Concatenate	(1024, 16, 16)	-
	Convolution (ReLU)	(512, 16, 16)	3x3
	Batch Normalization	(512, 16, 16)	-
	Convolution (ReLU)	(512, 16, 16)	3x3
	Batch Normalization	(512, 16, 16)	-
7	Transposed Convolution	(256, 32, 32)	2x2
	Concatenate	(512, 32, 32)	-
	Convolution (ReLU)	(256, 32, 32)	3x3
	Batch Normalization	(256, 32, 32)	-
	Convolution (ReLU)	(256, 32, 32)	3x3
	Batch Normalization	(256, 32, 32)	-
8	Transposed Convolution	(128, 64, 64)	2x2
	Concatenate	(256, 64, 64)	-
	Convolution (ReLU)	(128, 64, 64)	3x3
	Batch Normalization	(128, 64, 64)	-
	Convolution (ReLU)	(128, 64, 64)	3x3
	Batch Normalization	(128, 64, 64)	-
9	Transposed Convolution	(64, 128, 128)	2x2
	Concatenate	(128, 128, 128)	-
	Convolution (ReLU)	(64, 128, 128)	3x3
	Batch Normalization	(64, 128, 128)	-
	Convolution (ReLU)	(64, 128, 128)	3x3
	Batch Normalization	(64, 128, 128)	-
10	Convolution (Sigmoid)	(1, 128, 128)	lxl

**Table 7:** 3D U-Net++ model structure for tumor segmentation.

Layer	Type	Output Shape	Kernel Size
0	Input Layer	(4, 128, 128)	-
1	Convolution (ReLU)(d1)	(64, 128, 128)	3x3
	Convolution (ReLU)(d1)	(64, 128, 128)	3x3
	Max Pooling(p1)	(64, 64, 64)	2x2
	Batch Normalization(p1)	(64, 64, 64)	-
	Dropout(p1)	(64, 64, 64)	-
2	Convolution (ReLU)(d2)	(128, 64, 64)	3x3
	Convolution (ReLU)(d2)	(128, 64, 64)	3x3
	Max Pooling(p2)	(128, 32, 32)	2x2
	Batch Normalization(p2)	(128, 32, 32)	-
	Dropout(p2)	(128, 32, 32)	-
3	Convolution (ReLU)(d3)	(256, 32, 32)	3x3
	Convolution (ReLU)(d3)	(256, 32, 32)	3x3
	Max Pooling(p3)	(256, 16, 16)	2x2
	Batch Normalization(p3)	(256, 16, 16)	-
	Dropout(p3)	(256, 16, 16)	-
4	Convolution (ReLU)(d4)	(512, 16, 16)	3x3
	Convolution (ReLU)(d4)	(512, 16, 16)	3x3
	Max Pooling(p3)	(512, 8, 8)	2x2
	Batch Normalization(p3)	(512, 8, 8)	-
	Dropout(p3)	(512, 8, 8)	-
5	Convolution (ReLU)(d4)	(512, 8, 8)	3x3
	Convolution (ReLU)(d4)	(512, 8, 8)	3x3
6	Transposed Convolution(du 01)	(64, 128, 128)	3x3
	Concatenate[du 01,d1](du 01)	(128, 128, 128)	-
	Dropout(du 01)	(128, 128, 128)	-
	Convolution (ReLU)(c 01)	(64, 128, 128)	3x3
	Convolution (ReLU)(c 01)	(64, 128, 128)	3x3
7-1	Transposed Convolution(du 11)	(128, 64, 64)	3x3
	Concatenate[du 11,d2](du 11)	(256, 64, 64)	-
	Dropout(du 11)	(256, 64, 64)	-
	Convolution (ReLU)(c 11)	(128, 64, 64)	3x3
	Convolution (ReLU)(c 11)	(128, 64, 64)	3x3
7-2	Transposed Convolution(du 02)	(64, 128, 128)	3x3
	Concatenate[du 02,du 01,d1](du 02)	(256, 128, 128)	-
	Dropout(du 02)	(256, 128, 128)	-
	Convolution (ReLU)(c 02)	(64, 128, 128)	3x3
	Convolution (ReLU)(c 02)	(64, 128, 128)	3x3
8-1	Transposed Convolution(du 21)	(256, 32, 32)	3x3
	Concatenate[du 21,d3](du 21)	(512, 32, 32)	-
	Dropout(du 21)	(512, 32, 32)	-
	Convolution (ReLU)(c 21)	(256, 32, 32)	3x3
	Convolution (ReLU)(c 21)	(256, 32, 32)	3x3
8-2	Transposed Convolution(du 12)	(128, 64, 64)	3x3
	Concatenate[du 12,c 11,d2](du 12)	(384, 64, 64)	-
	Dropout(du 12)	(384, 64, 64)	-
	Convolution (ReLU)(c 12)	(128, 64, 64)	3x3
	Convolution (ReLU)(c 12)	(128, 64, 64)	3x3
8-3	Transposed Convolution(du 03)	(64, 128, 128)	3x3
	Concatenate[du 03,c 02,c 01,d1](du 03)	(256, 128, 128)	-
	Dropout(du 03)	(256, 128, 128)	-
	Convolution (ReLU)(c 03)	(64, 128, 128)	3x3
	Convolution (ReLU)(c 03)	(64, 128, 128)	3x3
9-1	Transposed Convolution(u 31)	(512, 16, 16)	3x3
	Concatenate[u 31,d4](u 31)	(1024, 16, 16)	-
	Dropout(u 31)	(1024, 16, 16)	-
	Convolution (ReLU)(c 31)	(256, 16, 16)	3x3
	Convolution (ReLU)(c 31)	(256, 16, 16)	3x3
9-2	Transposed Convolution(u 22)	(256, 32, 32)	3x3
	Concatenate[u 22,c 21,d3](u 22)	(768, 32, 32)	-
	Dropout(u 22)	(768, 32, 32)	-
	Convolution (ReLU)(c 22)	(256, 32, 32)	3x3
	Convolution (ReLU)(c 22)	(256, 32, 32)	3x3
9-3	Transposed Convolution(u 13)	(128, 64, 64)	3x3
	Concatenate[u 13,c 12,c 11,d2](u 13)	(512, 64, 64)	-
	Dropout(u 13)	(512, 64, 64)	-
	Convolution (ReLU)(c 13)	(128, 64, 64)	3x3
	Convolution (ReLU)(c 13)	(128, 64, 64)	3x3
9-4	Transposed Convolution(u 04)	(64, 128, 128)	3x3
	Concatenate[u 04,c 03,c 02,c 01,d1](u 04)	(320, 128, 128)	-
	Dropout(u 04)	(320, 128, 128)	-
	Convolution (ReLU)(c 04)	(64, 128, 128)	3x3
	Convolution (ReLU)(c 04)	(64, 128, 128)	3x3
10	Concatenate[c 01,c 02,c 03,c 04]	(256, 128, 128)	-
11	Convolution (Sigmoid)	(128, 128, 1)	1x1

### Web application UI

**Fig 8:**
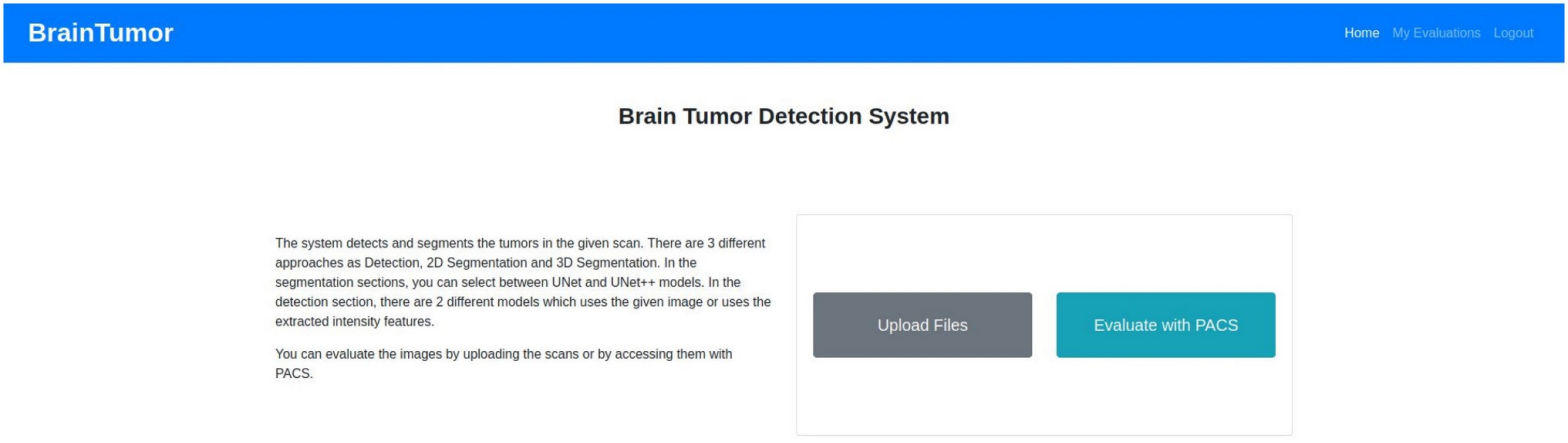
Home page.

**Fig 9:**
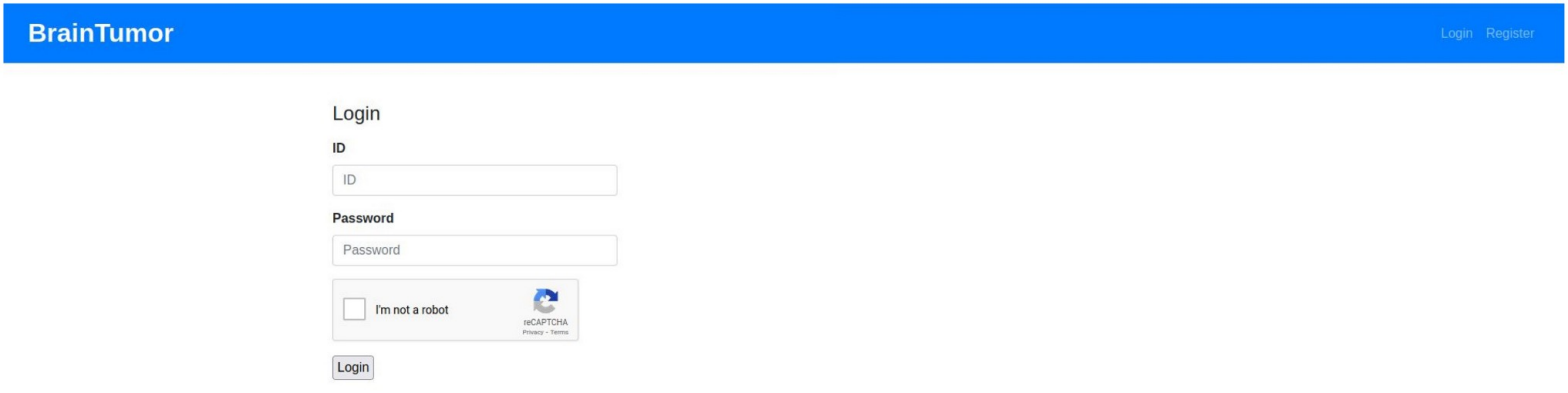
Login page.

**Fig 10:**
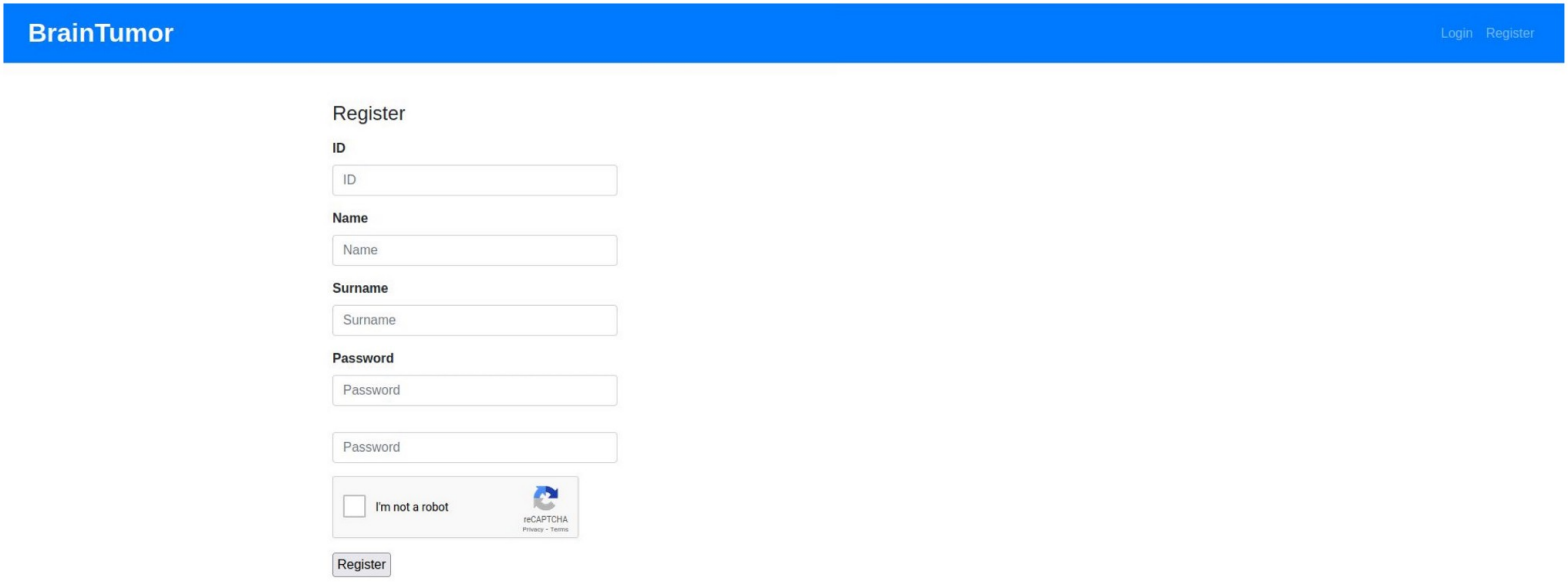
Registration page.

**Fig 11:**
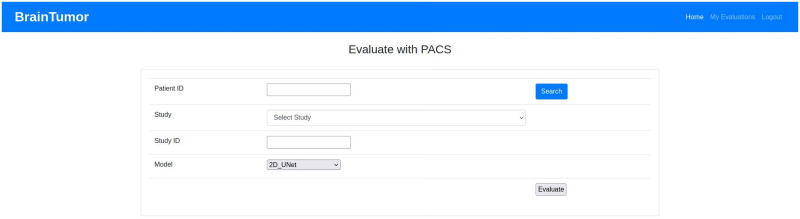
PACS page.

The web application for the proposed brain tumor detection and segmentation system can be browsed from the home page as shown in Fig 8. The home page includes a short description of the system and the options to either directly upload an image for evaluation or using the PACS to access medical images from hospitals. If the user is a registered user then they can login to the system with their ID and password as shown in Fig 9. Fig 10 shows the process for new users who can also register to the system with an ID, given name, surname and password to use the system. If the user wants to directly access the medical image from the hospital system, they can access the ‘Evaluate with PACS’ option from the homepage which leads to the PACS page as shown in Fig 11. The user can search with a valid patient ID and select the study type to choose a medical image. Then they can enter the study ID and choose the model they want to apply on the image. Finally, after entering all fields, they can click on the ‘Evaluate’ button to evaluate the image with the chosen DL model and get the results.

**Fig 12:**
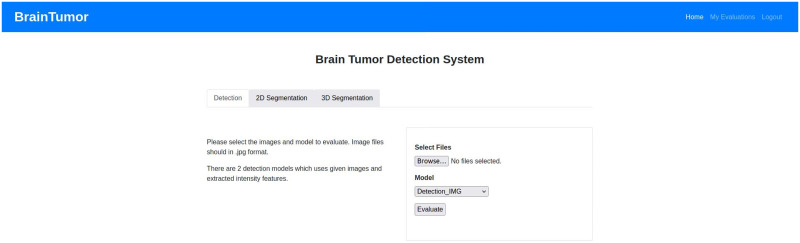
Tumor detection page.

**Fig 13:**
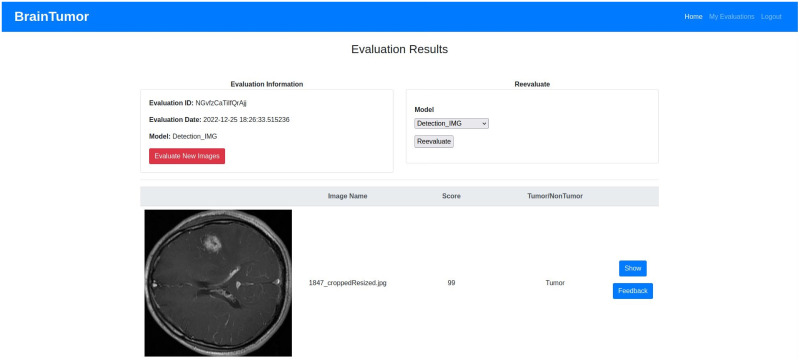
Tumor detection evaluation results page.

**Fig 14:**
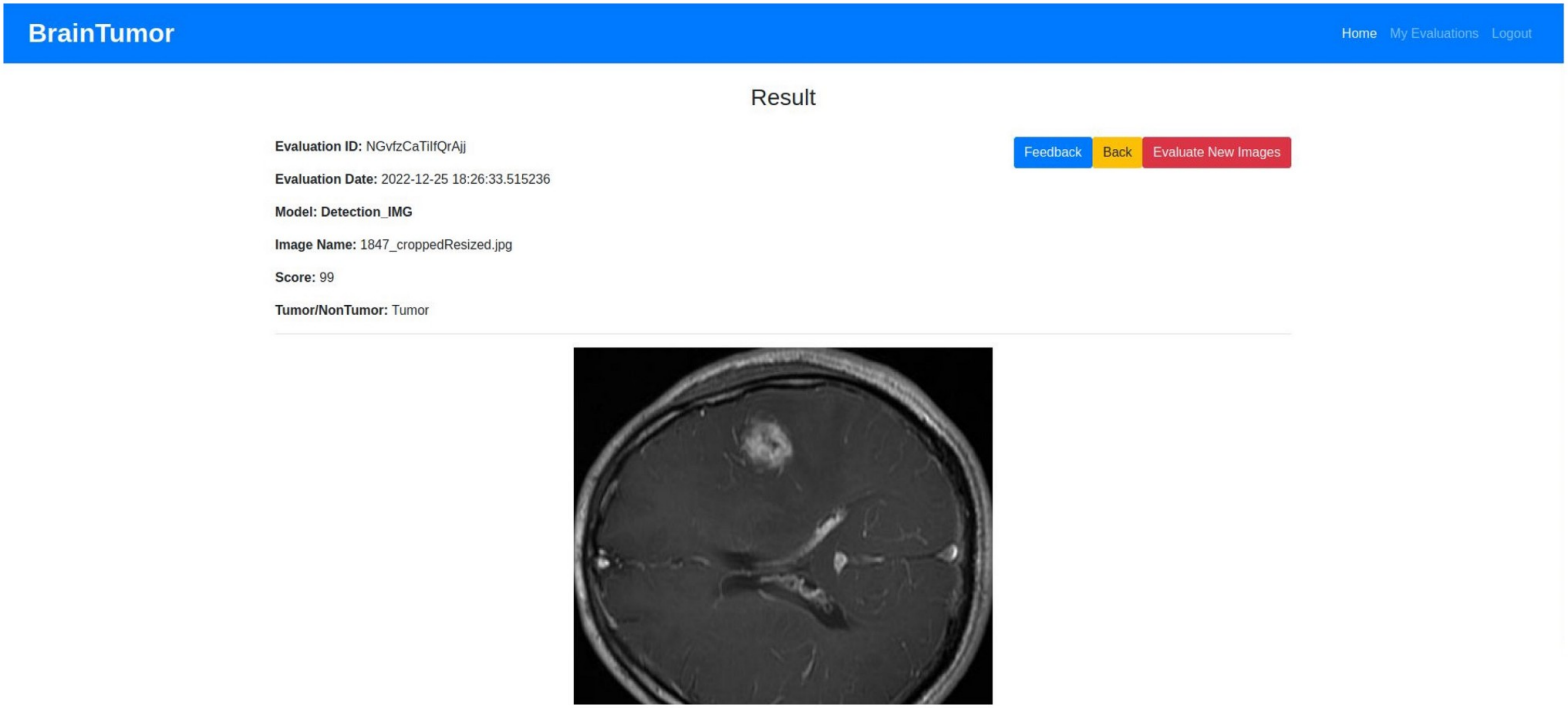
Tumor detection show result page.

**Fig 15:**
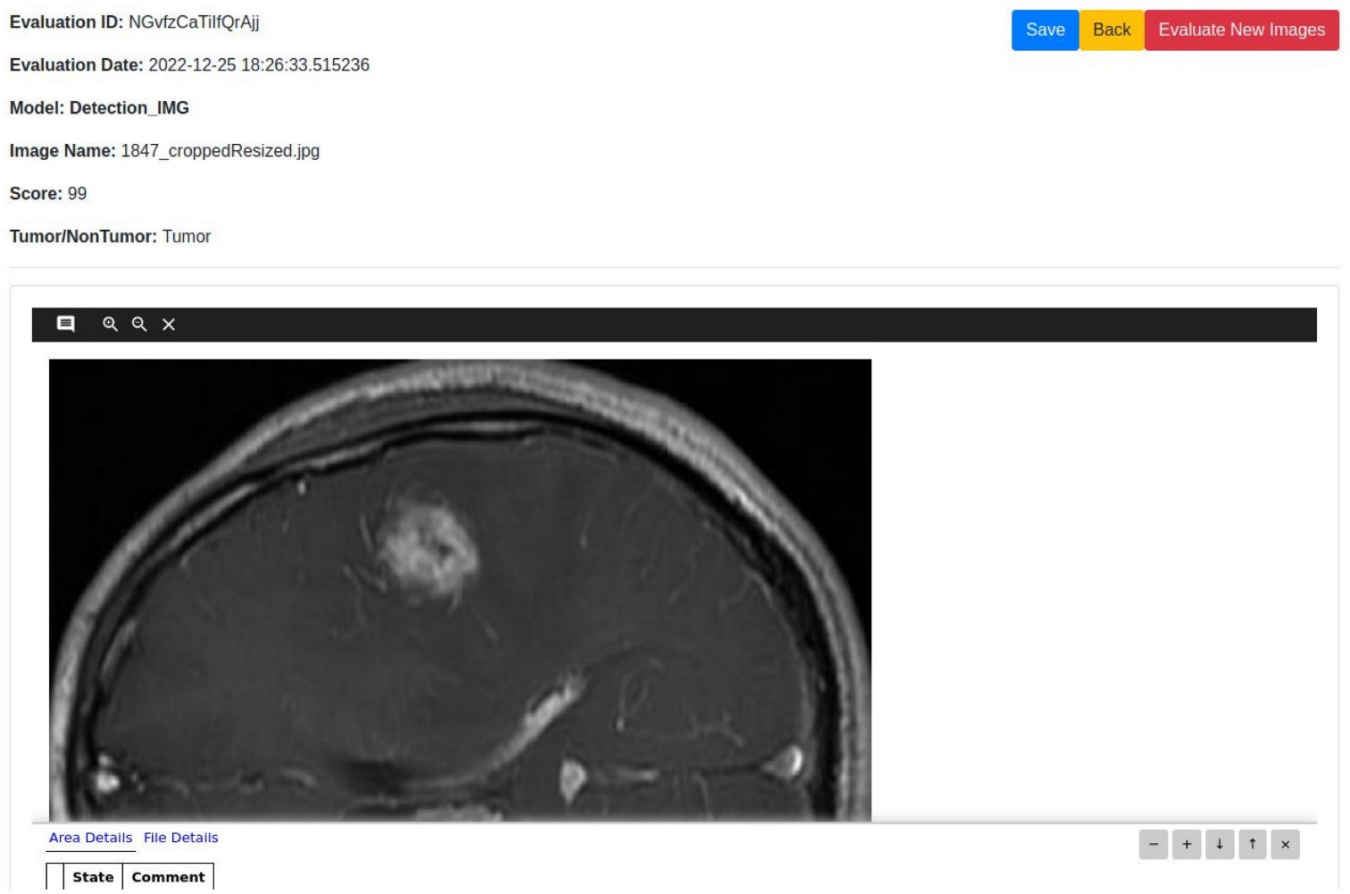
Tumor detection evaluation feedback page.

After the user logs in to the system, they have three options—‘Detection’, ‘2D Segmentation’ and ‘3D Segmentation’. If they choose ‘Detection’, they can upload the image (in .jpg or .png or .jpeg or .dcm formats) for applying the brain tumor detection models and the result shows whether there is a tumor present in the image or not as shown in Fig 12. After clicking on ‘Evaluate’, the chosen model is applied to the uploaded image and the prediction result is shown in the evaluation results as in Fig 13 with the decision (i.e. tumor or non-tumor) and the probability score of the prediction result. The user can click on ‘Show’ to check the result details in results as shown in Fig 14 and they can also provide feedback on the result using the ‘Feedback’ button. The feedback page is shown in Fig 15 and it allows the user to enter their feedback on the existence of tumor in the ‘State’ option and it allows them to include more details in the ‘Comment’ if necessary.

**Fig 16:**
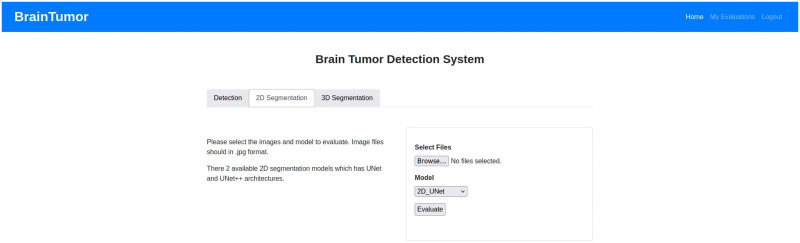
2D segmentation page.

**Fig 17:**
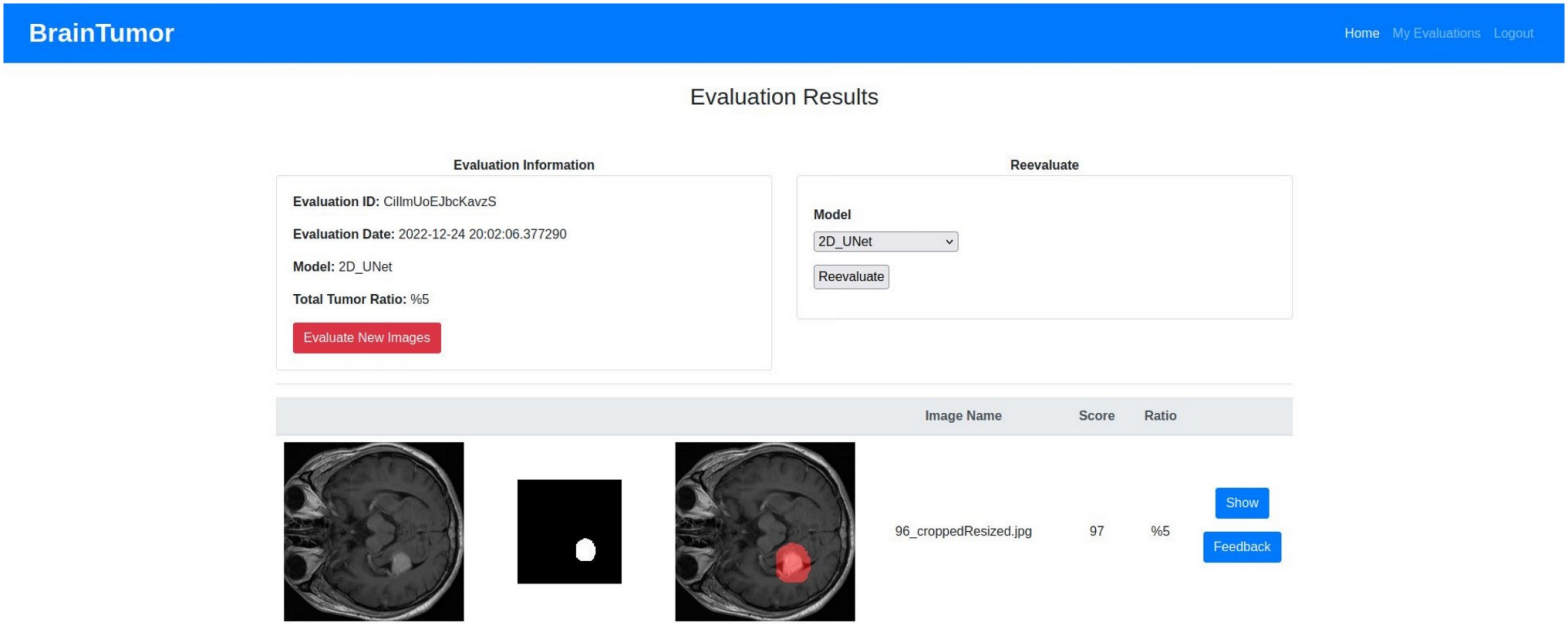
Evaluation results page.

**Fig 18:**
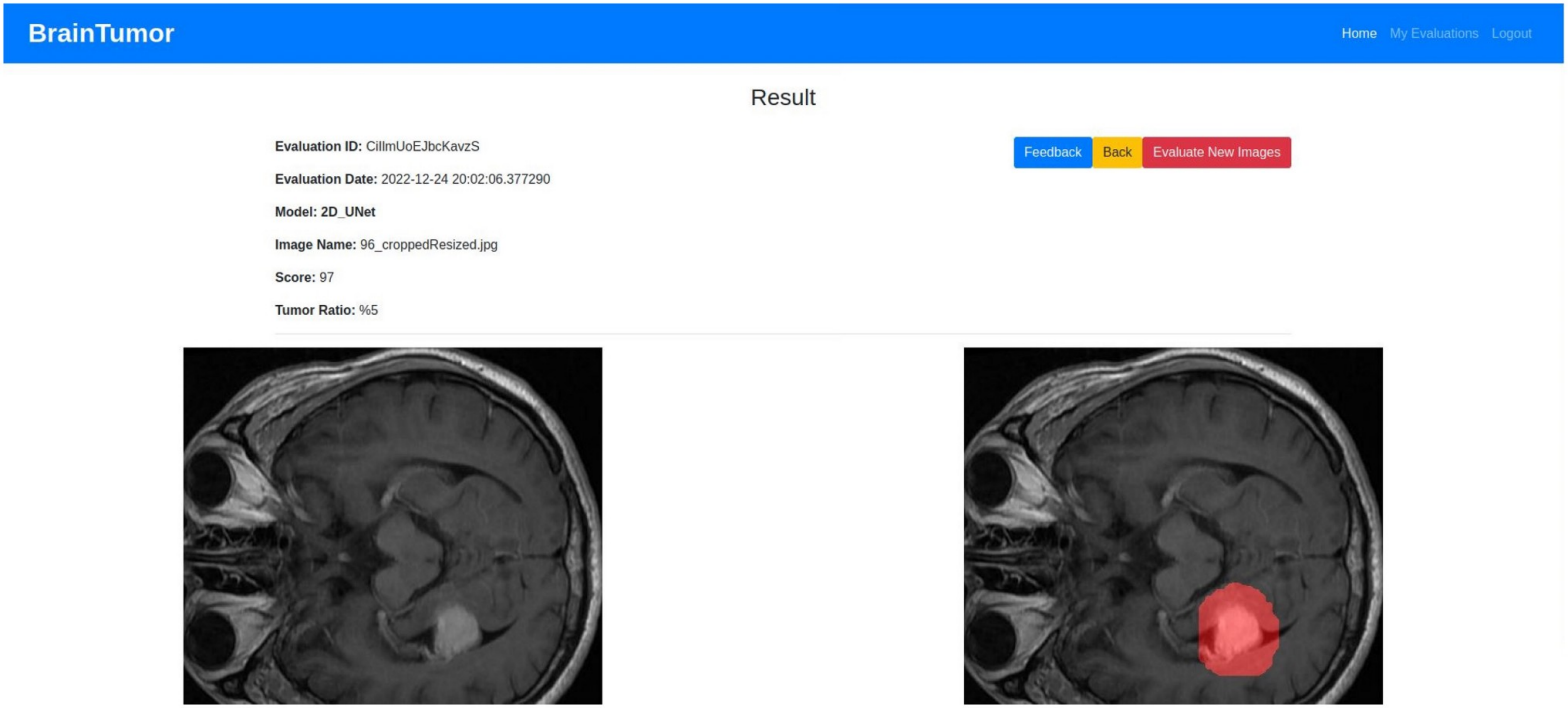
Show results page.

**Fig 19:**
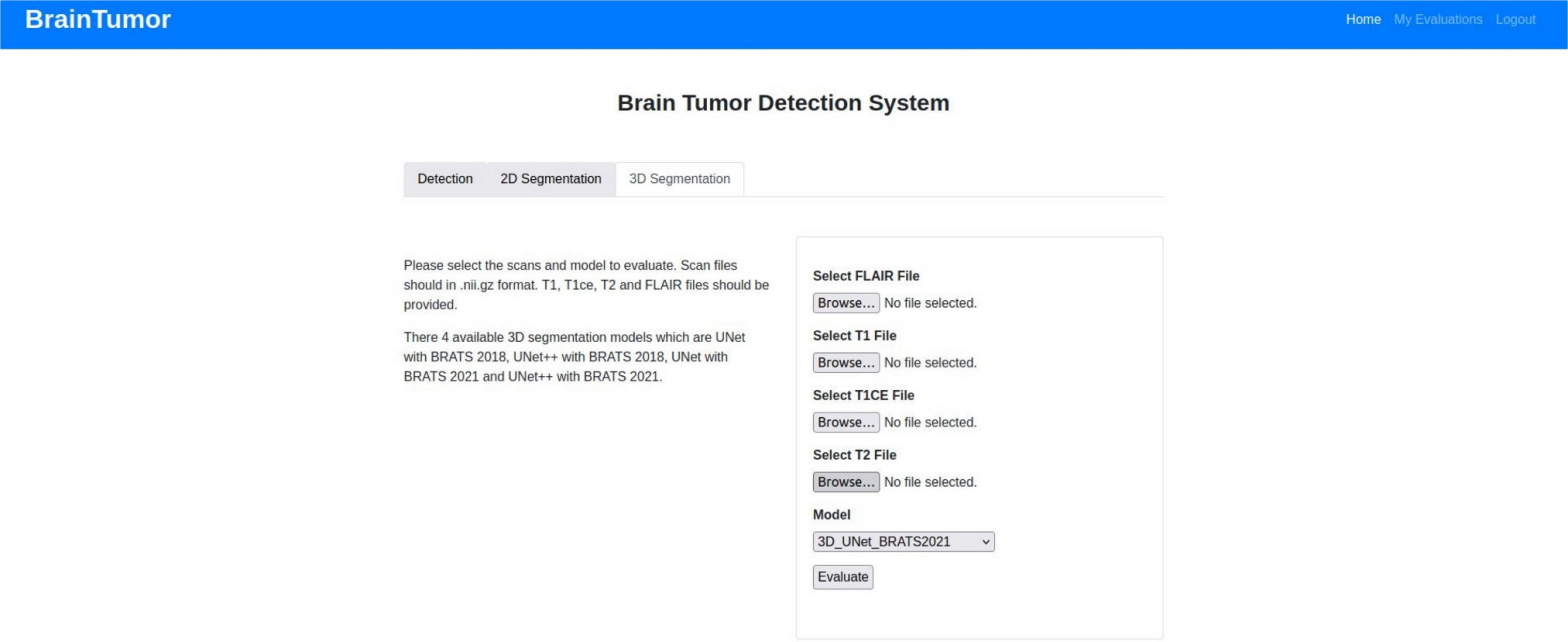
3D segmentation page.

**Fig 20:**
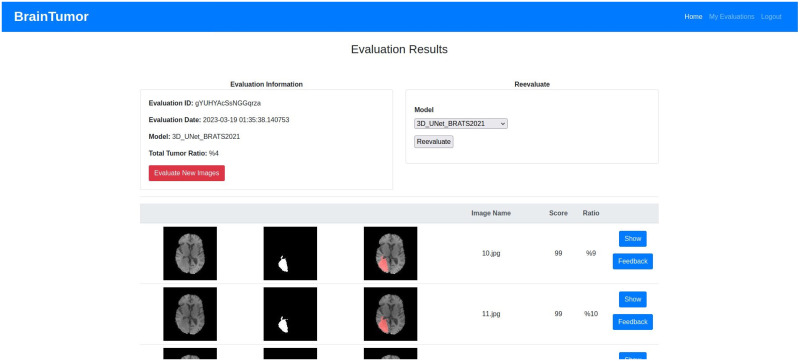
3D segmentation results page.

If the user chooses the ‘2D segmentation’ option, then they can upload the image (in .jpg or .png or .jpeg or .dcm formats) and choose one of the two DL models (i.e. U-Net or U-Net++) as shown in Fig 16. The evaluation results page shows the segmented tumor, the tumor area colored in red in the original image, the tumor to total brain area ratio and the segmentation confidence score. The user can evaluate another image, reevaluate the same image with another model or provide feedback on the evaluation as shown in Fig 17. They can click on the ‘Show’ button to see the results in details like Fig 18. The same approach can be followed for 3D segmentation. The user can go to the 3D segmentation option, upload the images and choose one of the two models for segmentation as shown in Fig 19. The only difference is that the 3D segmentation accepts four input files for all modalities (i.e. T1, T2, T1ce and FLAIR) in nifti (i.e. .nii or .nii.gz) formats. Fig 20 shows the results of 3D segmentation.

**Fig 21:**
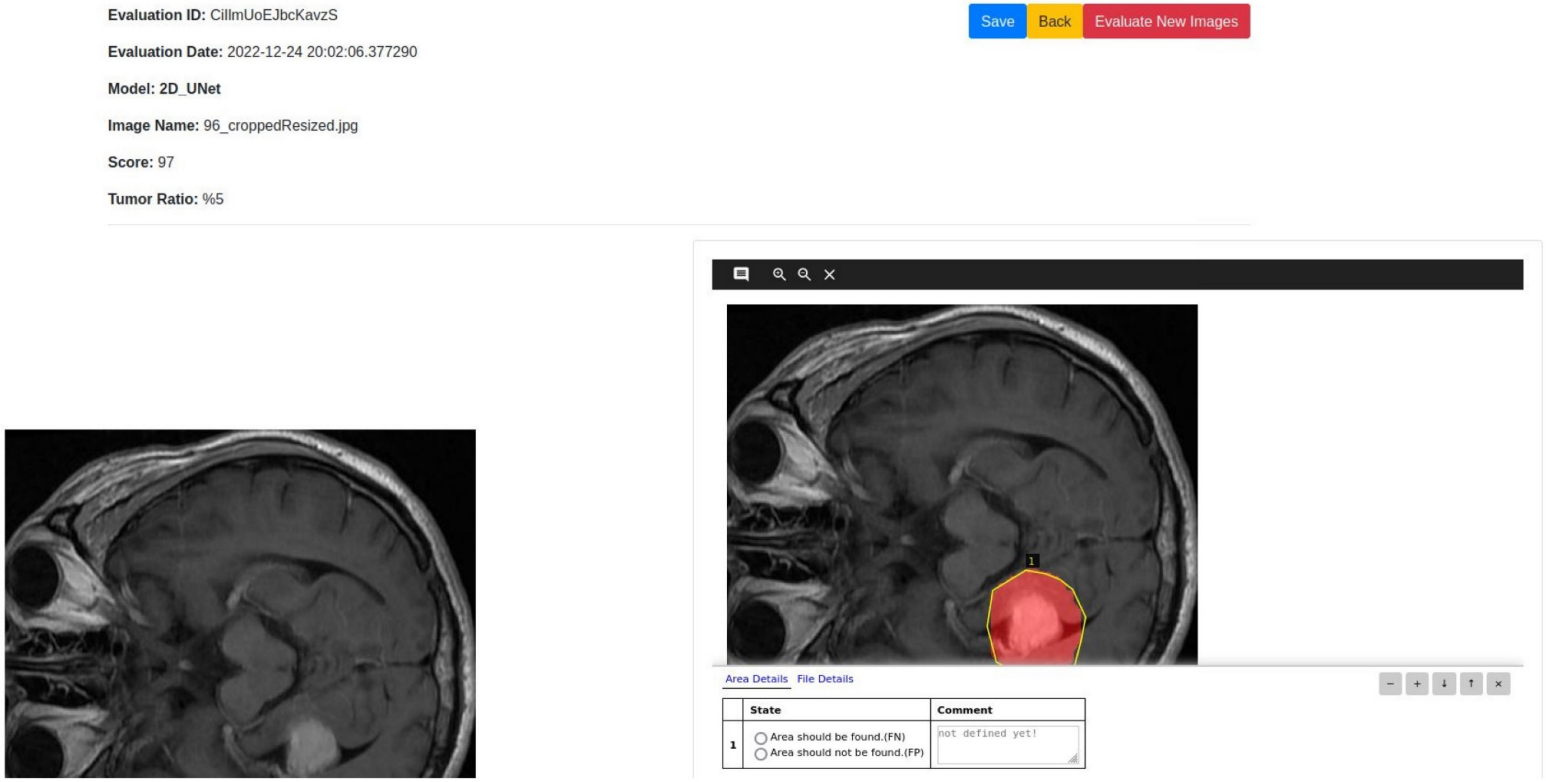
Feedback page.

**Fig 22:**
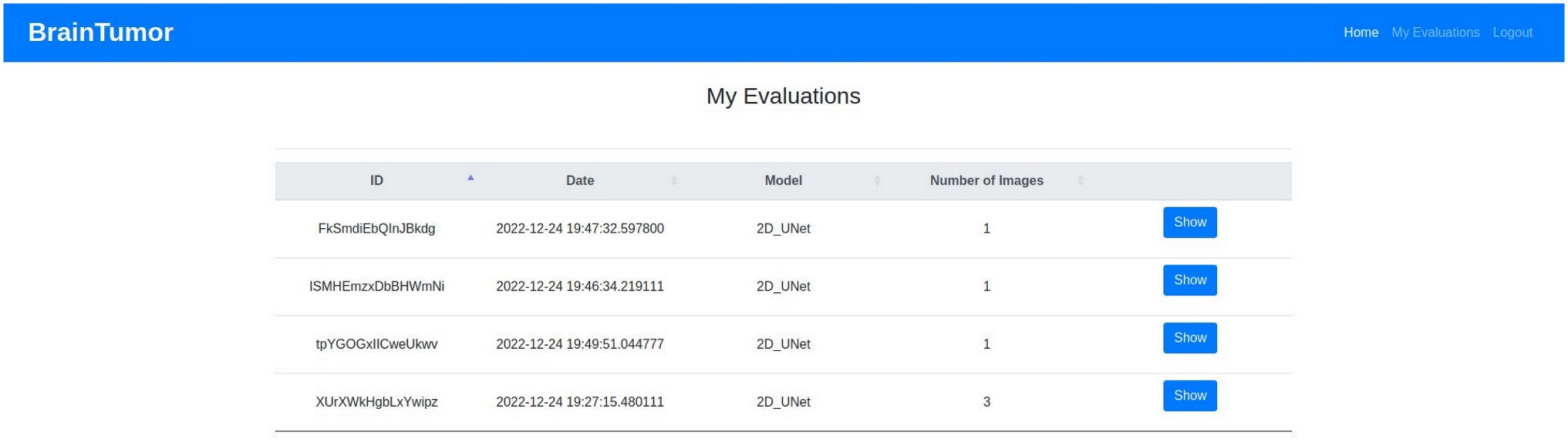
My evaluations page.

The user can provide feedback using the ‘Feedback’ option. They can mention the state of the result as ‘Area should be found (FN)’ for false negative outputs and ‘Area should not be found (FP)’ for false positive results. They can also provide or draw a contour of the tumor area in case the segmented area is not completely accurate. They can include any other comments they might have in the ‘Comment’ section as shown in Fig 21. The user can also see all evaluations they have executed on ‘My evaluations’ page as mentioned in Fig 22 with the evaluation ID, date and time of the evaluation, the models used and number of images used. They can also check the results from the ‘Show’ option.

### Results

The model accuracies for the detection and segmentation and the Dice scores for the segmentation models are computed as performance evaluation scores. Accuracy refers to the ratio of correctly predicted data against the total number of predicted data. Eq 1 shows the formula for the accuracy. The Dice score is calculated to define the similarity between two images and it is a popular performance metrics for image segmentation tasks. The Dice score (i.e. also known as Dice coefficient or Dice similarity coefficient or overlap index) represents the amount of overlap between the reference image (i.e. ground truth) and the output (i.e. segmented image). Let, P and Q be the output image and the ground truth image respectively. Then the Dice score can be calculated using Eq 2.

$$\begin{array}{c} \text{Accuracy}=\frac{\text{Total}\;\text{number}\;\text{of}\;\text{correct}\;\text{predictions}}{\text{Total}\;\text{number}\;\text{of}\;\text{predictions}} \end{array}$$ (1)

$$\begin{array}{c} \text{Dice}\;\text{score}\;\left(\text{P},\text{Q}\right)=\frac{2|\text{P}\cap \text{Q}|}{\left|\text{P}|+|\text{Q}\right|}. \end{array}$$ (2)

Table 8 shows the tumor detection performance evaluation for MRI image input and image features inputs for MRI classification into tumor and non-tumor classes. The model with direct MRI image input achieved more than 95% accuracy at the training phase and more than 82% accuracy at the validation phase. The accuracy at the testing phase declined to 70%, but that was still a high accuracy for tumor detection. The results for the tumor detection with each feature separately and the combination of all features collected from the images showed a different outcome. The accuracy for the training and validation models varied between 68% to 80% where sometimes the validation accuracy was higher and other times the training accuracy was higher. But in all cases, the prediction accuracy for the testing data (i.e. new data that was not used during training or validation) was much higher and varied between 80% to 93%. The features separately collected from the images were able to classify the tumor/non-tumor images better than the features automatically generated from image by the CNN model.

**Table 8:** Tumor/Non-tumor detection with image and image features.

Input	Prediction Accuracy	Training Accuracy	Validation Accuracy
MRI	76.47	89.85	75.76
Intensity Features	88.24	71.86	75.12
GLCM Features	84.31	79.37	78.05
DWT Features	82.35	75.40	77.46
Other Features	80.39	73.52	68.29
All Features	92.16	69.70	76.41

**Fig 23:**
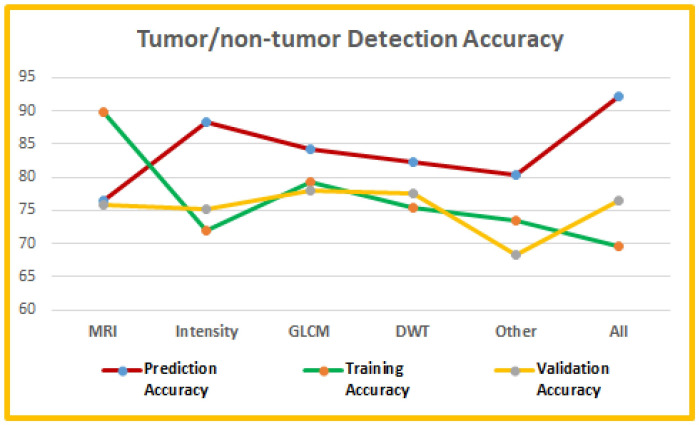
Performance comparison for tumor/non-tumor detection task.

Although the training and validation accuracies for the model that directly used MR image as input were visibly higher than the feature based outputs, the prediction accuracy shows that the CNN models with feature inputs were able to predict the tumorous and non-tumorous images with at least more then 4% to 16% accuracy. The performances of the detection models with extracted feature inputs were better than direct image input as the amount of data was not sufficient and the quality of all input images were not consistent. Some of the input images have visible distinguishing characteristics (i.e., edges, boundaries, solid areas, etc.) between the tumor area and the rest of the image, but that was not the case for all images. Hence, the prediction accuracy of the CNN model using the images as inputs for the detection task was slightly lower than the prediction accuracy of the model with separate and/or all features. The model was able to predict the existence or absence of a brain tumor from all features more accurately compared to separate feature sets. For this reason, the trained model for image detection with all features was added as one of the user choices for classifying the image. The prediction accuracy comparisons also show that the intensity features were able to contribute to the prediction task better than other separate sets of features and that is why the intensity feature based prediction model was also added as an option to the UI. Fig 23 shows the comparison in a graph where the red line represents the prediction accuracy, the green line represents the training accuracy and the yellow line refers to the validation accuracy for the MRI CNN features, intensity features, GLCM features, DWT features, other features and all (i.e. intensity, GLCM, DWT, other) features.

**Fig 24:**
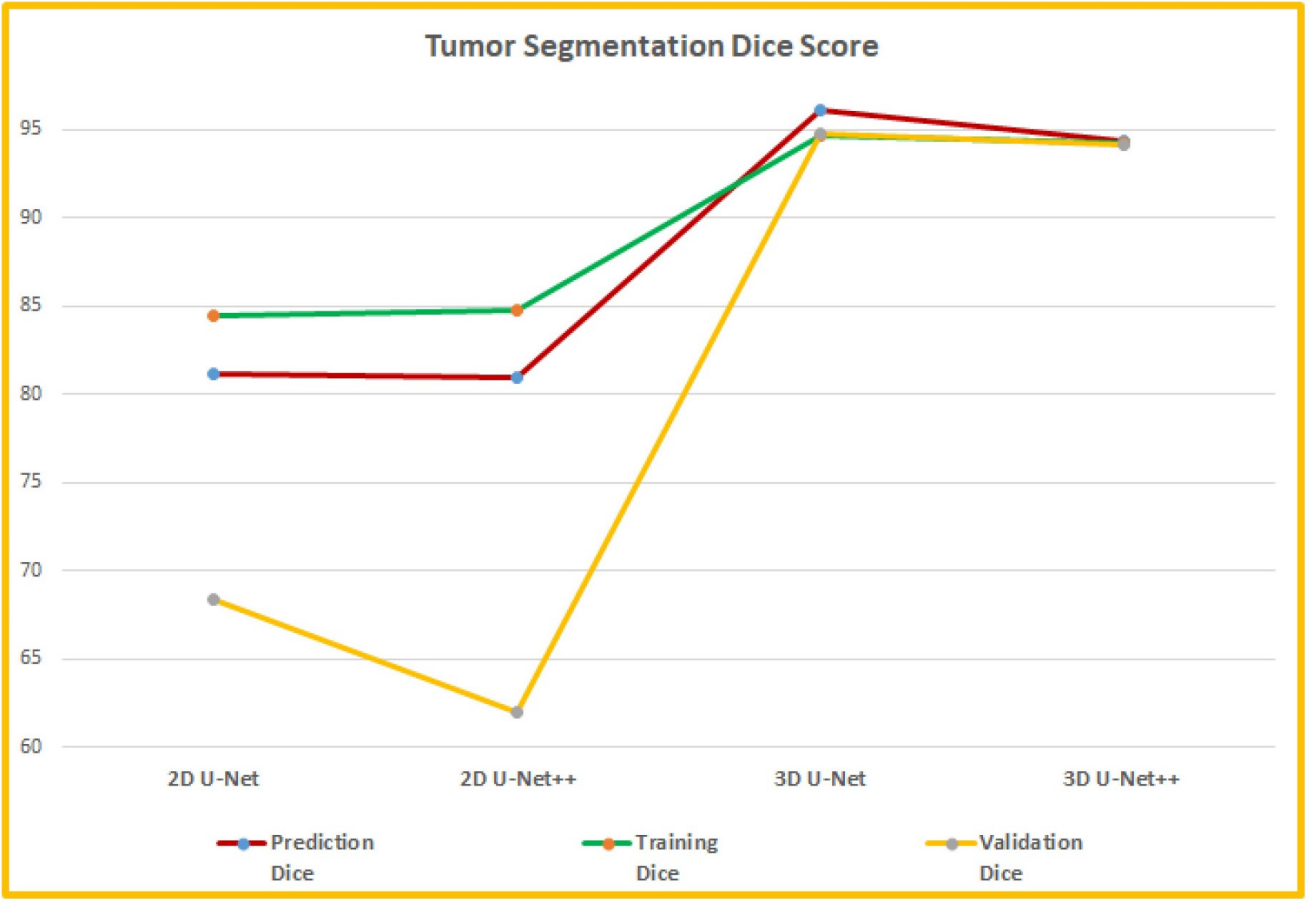
Performance comparison for tumor segmentation task.

**Fig 25:**
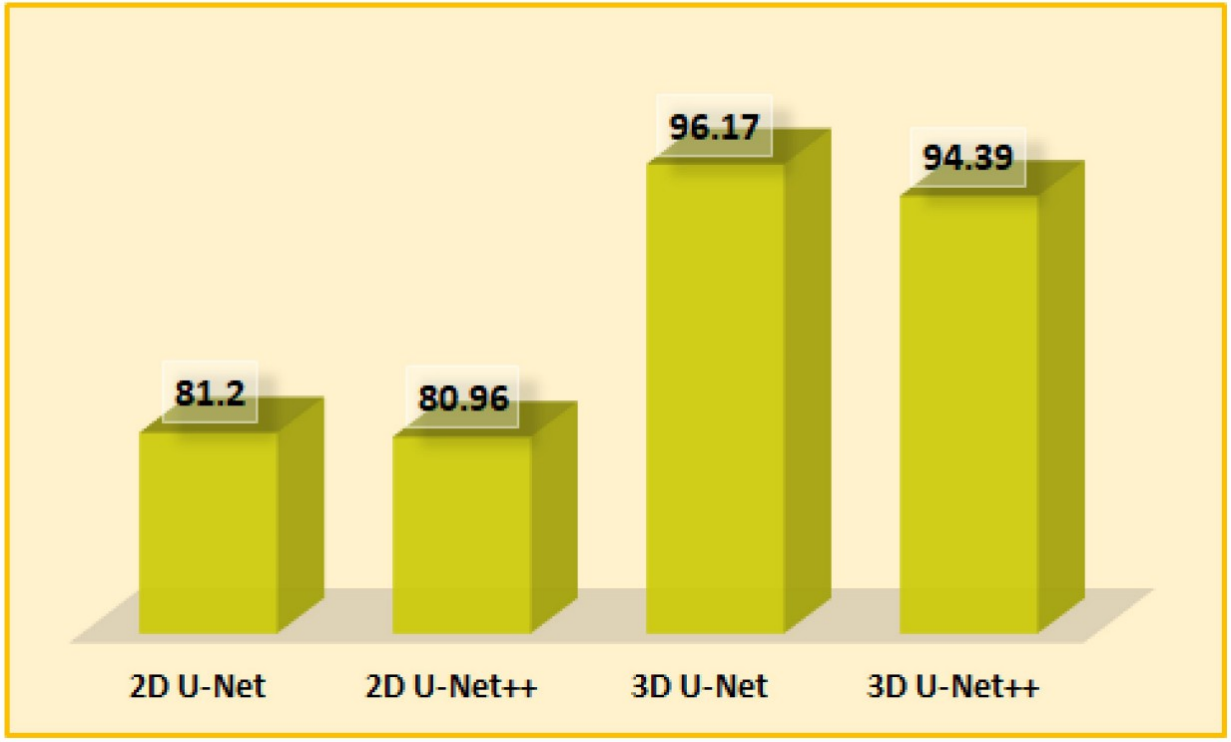
Performance comparison for 2D and 3D tumor segmentation (average prediction Dice scores).

Table 9 and Fig 24 show performance evaluations for the 2D and 3D MRI segmentations with U-Net and U-Net++ models. The 2D segmentations achieved more than 84% and 61% training Dice score and validation Dice scores respectively. The prediction Dice scores on the test dataset were higher than the validation scores and varied from 80% to almost 82%. The 3D segmentation models achieved higher Dice scores for training, validation and testing data and all scores were higher than 90% attaining more than 96%. The 3D U-Net models achieved almost same to 2% higher Dice scores for the test datasets compared to the training and validation whereas the 3D U-Net++ models had almost no difference between training, validation and testing data. The results showed that the U-Net models performed slightly better in tumor segmentation than the U-Net++ models for both 2D and 3D data. Although the training and validation Dice scores for most cases were very similar and the prediction Dice scores showed the differences between the models more clearly for both 2D and 3D data, the Dice scores for all implemented models were comparable to DL-based brain tumor segmentation. Fig 25 shows a more comprehensive plot diagram based on the average Dice scores of the test data prediction of the brain tumor segmentation models. The bar plot represents the performance differences between the 2D and the 3D models which varied between 14% to 16% showing the 3D models performed better. Another interesting observation on the U-Net and U-Net++ model performances was the similarity between them. Although the U-Net model performed better than the U-Net++ in both 2D and 3D images, the difference was only less than 2%.

**Table 9:** 2D and 3D tumor segmentation.

Model	Prediction Dice	Training Dice	Validation Dice
2D U-Net	81.20	84.48	68.37
2D U-Net++	80.96	84.77	61.92
3D U-Net	96.17	94.68	94.74
3D U-Net++	94.39	94.28	94.14

The resulting differences between the 2D DNNs and 3D DNNs can be caused by the differences in the 2D and 3D datasets. The 2D dataset contains 3064 t1-weighted 2D MRIs combining images of three different anatomical planes. They also have different intensities for tumor and non-tumor regions and these intensities are not consistent. In some images the tumor region intensity is higher than the rest of the image, in some images it is lower, and it is not distinguishable in others. The 2D dataset has images that include the skull, brain and tumor and the differences between these are not prominent. The 3D dataset is a processed benchmark dataset containing all axial view 3D MRIs with skull-stripped brain images and the tumor regions are distinguishable in almost all files. The 2D slices collected from the 3D files also provide more specific and consistent information on the tumor region. Hence, the tumor segmentation from the 3D dataset with U-net and U-Net++ achieved noticeably higher performances than U-Net and U-Net++ on the 2D dataset. Table 10 shows some comparisons between the Dice scores of whole tumor segmentations between the implemented models in this research and baseline U-Net [84], U-Net++ [51] for BRATS 2021 dataset. The results show that the implemented models in this research achieved almost 3% to 10% higher Dice scores in segmenting the whole tumor from the BRATS 2021 dataset for both U-Net and U-Net++ models. The hyperparameter tuning and hybrid loss function improved the performances of the implemented model by a large scale compared to baseline models.

**Table 10:** Comparison of whole tumor segmentation with BRATS 2021.

Model	Dice Scores
3D U-Net [84]	93.24
3D U-Net (ours)	96.17
3D U-Net++ [51]	84.93
3D U-Net++ (ours)	94.39

**Fig 26:**
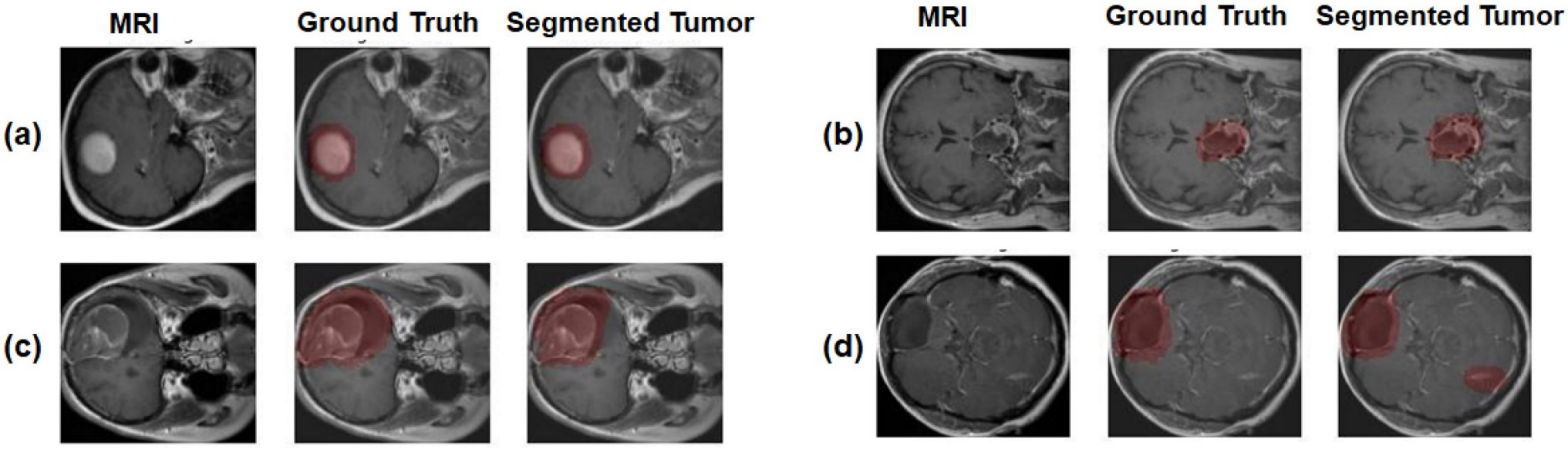
Sample segmentation outputs for 2D U-Net segmentation.

**Fig 27:**
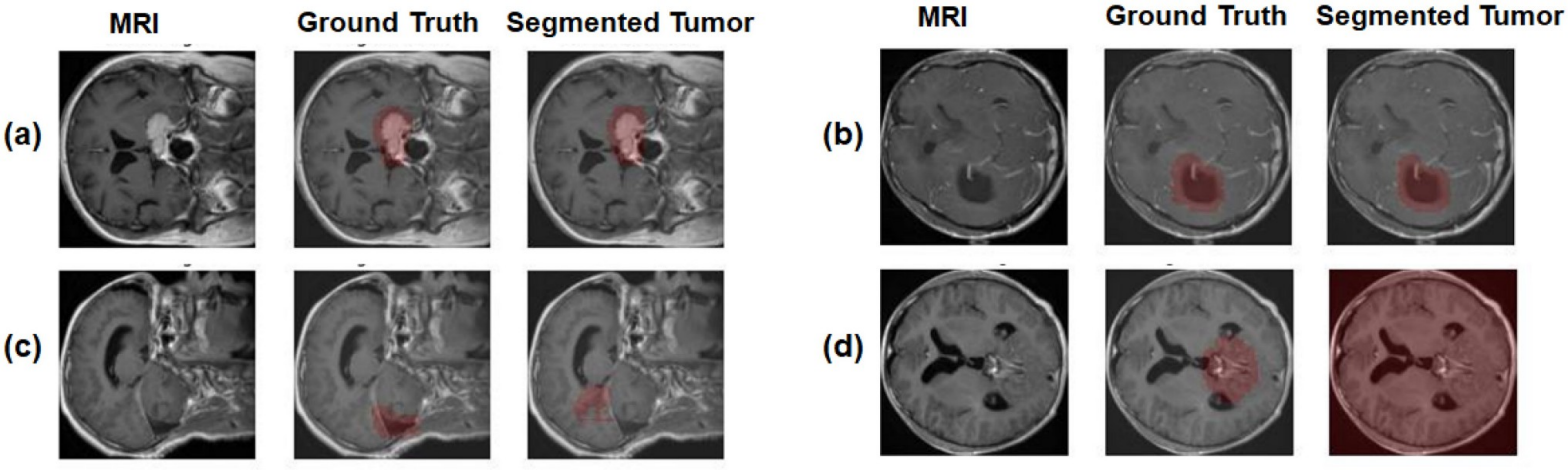
Sample segmentation outputs for 2D U-Net++ segmentation.

**Fig 28:**
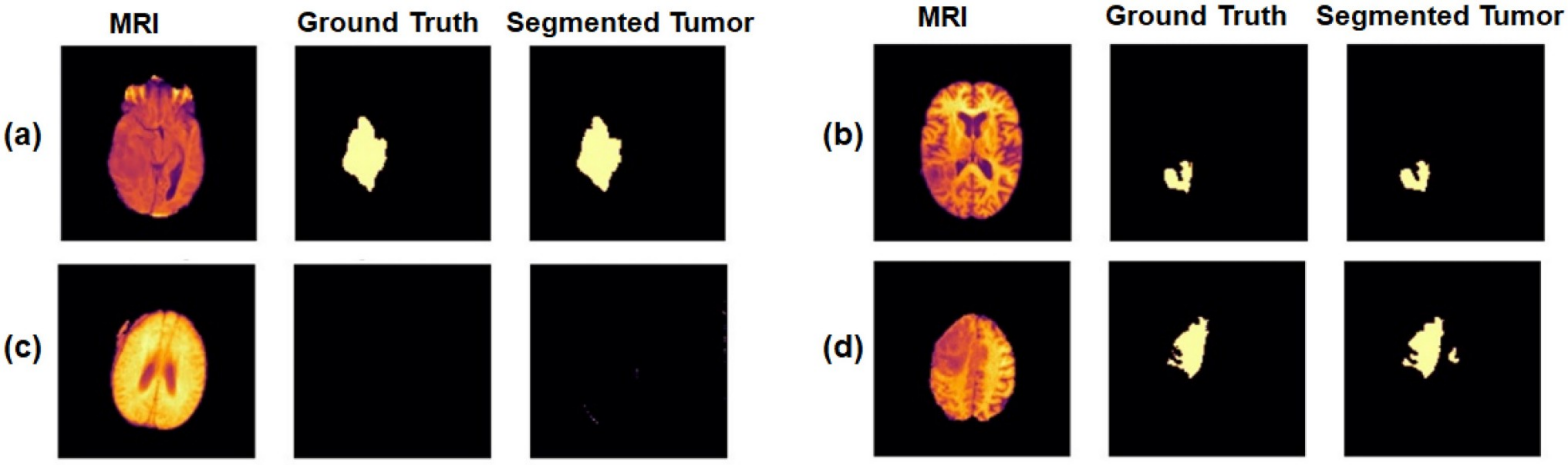
Sample segmentation outputs for 3D U-Net segmentation.

**Fig 29:**
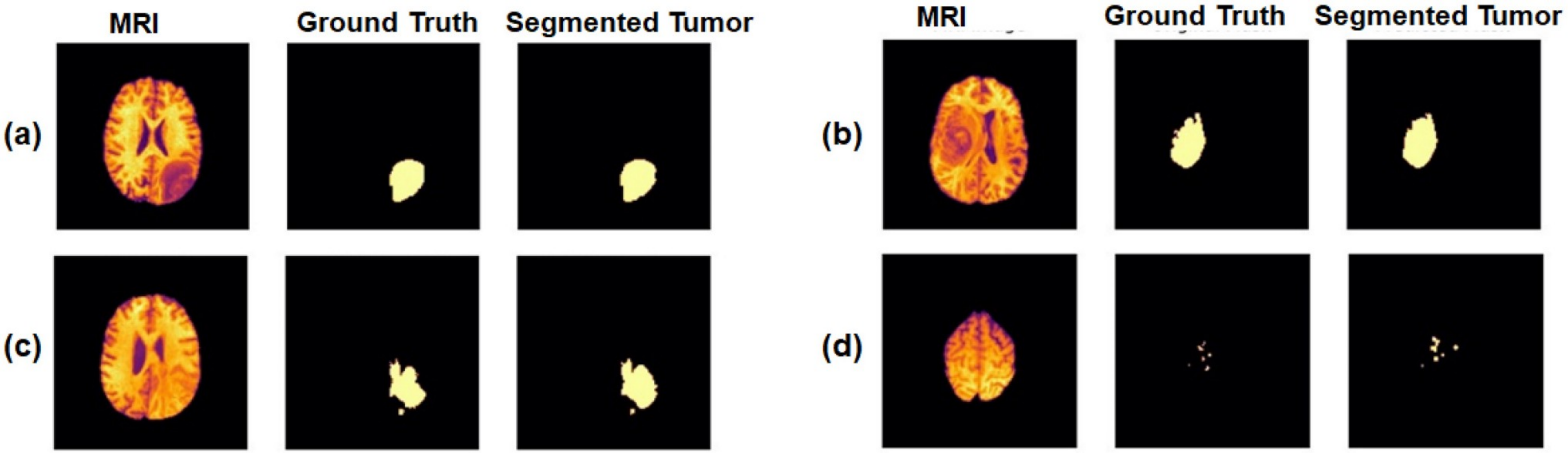
Sample segmentation outputs for 3D U-Net++ segmentation.

Figs 26 and 27 show some sample input MRIs, the tumor and predicted tumor marked in red for 2D U-Net and 2D U-Net++ respectively. Similarly, Figs 28 and 29 show some sample input MRIs, the tumor and predicted tumor for 3D U-Net and 3D U-Net++ respectively. Although most of the segmentation outputs were able to segment the tumors properly, there were also some cases where the models were i) not able to detect the tumor, ii) detected tumor area even if there were not any tumors, iii) segmented the tumor and some extra area, iv) segmented a part of the tumor area. Cases i and ii were the worst case scenarios where the models were unable to focus on the tumor region of the test images. For example, in Fig 26, (a) and (b) show proper tumor area segmentation, whereas (c) shows some missing part in the segmented tumor compared to the ground truth and (d) shows some extra region at the opposite side of the brain segmented as tumor area. The examples in 2D U-Net++ show some more variations. Fig 27 shows proper segmentation in (a) and (b). But the segmentation example in (c) shows that the model could not detect the tumor area and detected another nearby but non-overlapping area as the tumor. The example in (d) shows one of the worst case scenarios, where the model detected the complete image as the tumor which is obviously incorrect. These examples support the Dice score results showing that the 2D U-Net model performed better in tumor segmentation compared to the 2D U-Net++ model. Figs 28 and 29 also provided similar results as the Dice scores. Both 3D segmentation models clearly provided better segmentation than the 2D models in most cases. The images show some slices from the 3D test images with their segmentations and like their Dice scores, the 3D U-Net segmented the tumors more accurately than the 3D U-Net++ model. Fig 28 (a) and (b) show that the segmented tumor area are exactly same as the ground truth. (c) shows that although there was no tumor in the MRI, the segmentation output shows very few nearby pixels at the lower left part of the image as the tumor. The 3D U-Net model segmented a small nearby area as the tumor as well with the original tumor in (d). Finally, Fig 29 (a) shows an accurate tumor segmentation output of 3D U-Net++, but the segmented tumor regions in (b) and (c) are slightly more rounder at the edges including few more nearby pixels as part of the tumors. So, for these two examples, the model detected a very similar but slightly bigger supersets of the tumor area pixels. On the other hand, the pixels present in the segmented output of (d) are mostly different than the ground truth pixels. Although there are few overlaps between the pixels of ground truth and segmented output, mostly the segmented output provided different pixels from similar area of the image as tumor. Each test dataset had hundreds of test images and corresponding outputs and these are just some examples. But these examples show most of the variations the test data showed for the complete test outputs for all 2D and 3D segmentation models.

## Conclusion

Medical image analysis is a popular non-invasive way of diagnosing diseases an hence helping medical professionals in their professional assessments. Researchers from various fields have been trying to improve this analysis process applying different methods to develop more accurate automated processes and systems using medical images from different organs and body parts. As brain is the most complex organ that controls most of the functionalities of our bodies, neurological disease analysis from medical images of brain (i.e. MRI, CT, PET etc.) is a well-explored research area. In this paper, we propose a complete web application to detect the existence of brain tumor and to segment the tumor area from medical images like brain MRIs to provide a primary and precise scanning phase to help the medical professionals. The proposed web application produces a complete automated system to upload brain medical images, analyze the uploaded images with different types of operations implemented by DL models and to allow feedbacks from medical professionals to be incorporated in the future training of the models.

The web application provides the users to directly upload medical images or they can use existing medical images from hospital databases with PACS and apply three types of operations—tumor detection, 2D tumor segmentation and 3D tumor segmentation. The tumor detection can be used with image or image features and CNN models process the inputs to generate a decision—tumor or no tumor with a probability score to show the accuracy of the prediction. Both 2D and 3D tumor segmentation can be used to upload 2D or 3D brain images and either U-Net or U-Net++ model can be chosen to apply the segmentation. The segmentation results show the segmented tumor, the ratio of the tumor area and the confidence score of the tumor segmentation process. The tumor detection for some features achieved more than 90% accuracy and the segmentation models achieved around 96% Dice scores for few models. The application also allows a feedback option for healthcare professionals to provide their feedbacks on the detection and segmentation to reduce the limitations of the results with text inputs, contouring inputs and checkbox inputs. The current application have some restrictions on the input image types for each operation due to the training of the DL models. The system works with few popular medical image based DL models like CNN, U-Net and U-net++ models. But the architecture of the application allows the possibility of adding any detection or segmentation models needed. So, possible future works for the proposed system would be to extend the system to allow any images of any dimension and format, adding more new DL models as options for the users to apply on the images and including more detail properties of the tumors in the results. Analyzing the MRIs more to detect and segment separate tumor tissues, computing various tumor features (i.e. spatial, biological, etc.), tumor/cancer severity prediction will be added in future extensions of this research.
